# Speed Dating with Voice User Interfaces: Understanding How Families Interact and Perceive Voice User Interfaces in a Group Setting

**DOI:** 10.3389/frobt.2021.730992

**Published:** 2022-01-14

**Authors:** Anastasia K. Ostrowski, Jenny Fu, Vasiliki Zygouras, Hae Won Park, Cynthia Breazeal

**Affiliations:** ^1^ Media Lab, Massachusetts Institute of Technology, Cambridge, MA, United States; ^2^ Information Science, Cornell University, Ithaca, NY, United States

**Keywords:** voice-user interface (VUI), social robot, emotional engagement, social embodiment, trust, behavioral analysis, small group, competence

## Abstract

As voice-user interfaces (VUIs), such as smart speakers like Amazon Alexa or social robots like Jibo, enter multi-user environments like our homes, it is critical to understand how group members perceive and interact with these devices. VUIs engage socially with users, leveraging multi-modal cues including speech, graphics, expressive sounds, and movement. The combination of these cues can affect how users perceive and interact with these devices. Through a set of three elicitation studies, we explore family interactions (*N* = 34 families, 92 participants, ages 4–69) with three commercially available VUIs with varying levels of social embodiment. The motivation for these three studies began when researchers noticed that families interacted differently with three agents when familiarizing themselves with the agents and, therefore, we sought to further investigate this trend in three subsequent studies designed as a conceptional replication study. Each study included three activities to examine participants’ interactions with and perceptions of the three VUIS in each study, including an agent exploration activity, perceived personality activity, and user experience ranking activity. Consistent for each study, participants interacted significantly more with an agent with a higher degree of social embodiment, i.e., a social robot such as Jibo, and perceived the agent as more trustworthy, having higher emotional engagement, and having higher companionship. There were some nuances in interaction and perception with different brands and types of smart speakers, i.e., Google Home versus Amazon Echo, or Amazon Show versus Amazon Echo Spot between the studies. In the last study, a behavioral analysis was conducted to investigate interactions between family members and with the VUIs, revealing that participants interacted more with the social robot and interacted more with their family members around the interactions with the social robot. This paper explores these findings and elaborates upon how these findings can direct future VUI development for group settings, especially in familial settings.

## 1 Introduction

Increasingly, technology is being developed to engage with us in seemingly more natural communicative ways resulting in users responding by interacting socially with devices such as voice user interfaces (VUIs) (Nass et al., 1994; Reeves and Nass, 1996). This has been a sustained interest for the human-robot interaction (HRI) community especially when applied to small groups and collaborative, multi-user spaces (Jung et al., 2017; Cambre and Kulkarni, 2019). VUIs allow users to interact vocally with the device, promoting a more “natural” interface than those that do not allow voice interaction. This includes devices such as smart speakers and social robots. Since VUIs can be found in homes, museums, airports, and malls to name a few places (Burgard et al., 1999; Fong et al., 2003; Sabanovic et al., 2006; Lee et al., 2010), it is quite common for these devices to interact with small groups of 2-6 people. Our work contributes to the growing body of HRI research on small groups of people interacting with one or more VUIs. Specifically, our work is formatted as an adapted “speed dating” format (Zimmerman and Forlizzi, 2017) elicitation study to explore how small groups, in our case families, interact with and perceive three commercially available VUI agents in a small group multi-agent format. In this paper, we propose a speed dating scenario to mimic likely first encounters of families trying out different VUI agents together. Such multi-agent format either mimics a situation where families or other small groups of people comparing voice agents such as in a retail scenario where potential users are buying voice agents, or a family trying out the new device together when it is delivered to their home. Shopping for VUI devices leads to integrating the technology in the home for family use, so it is only natural that we discuss them together. The speed dating approach allows two different types of analysis. First, we can dissect users’ interactions and behaviors with commercially available VUIs. Second, it provides a foundation for users to develop a mental model of the VUIs that researchers can leverage to understand users’ initial perceptions of these devices through additional measures and tools.

The motivation for this study is grounded in our earlier work (Singh, 2018) where we observed how people from children to older adults interact with different types of VUI devices (i.e., smart speakers and social robots). For instance, we have observed users engage and use social robots (Jibo) more than smart speakers (Amazon Echo and Google Home). However, our prior work did not strictly investigate or provide insights as to why these differences were observed. In a series of three studies presented in this paper focused on social embodiment, we iterate upon the elicitation study speed dating format to provide insights into user interactions and perceptions of commercially available VUIs, the robustness of these results, and demonstrate how studies can be iterated upon and refined to support replicability of concepts. The first study was our initial exploration into how users interact with and perceive three commercially available devices without any modifications to their commercial default settings. In this study, families interacted with Jibo (wake word: “Hey Jibo”), Amazon Echo (wake word: “Hey Alexa”), and Google Home (wake word: “Hey Google”). In the second study, we investigated the same three VUIs and primed participants to consider the manufacturer and company that built and designed the device. We adapted the wake words to emphasize the company (i.e., “Hey Jibo,” “Hey Amazon,” “Hey Google”). In the third study, we further modified the arrangement to create a spectrum of social embodiment with three VUIs. Participants interacted with Jibo (most socially embodied; wake word: “Hey Jibo”), Amazon Echo Spot modified with a rotating flag as an additional attention mechanism (middle socially embodied; wake word: “Hey Alexa”), and Amazon Echo Show (least socially embodied; wake word: “Hey Computer”). Each study revealed strikingly similar result patterns, demonstrating the impact of concepts such as embodiment and social presence on user interaction and perception.

The collection of studies presented here are the first example to our knowledge of these methods applied to commercial VUI agents small group multi-agent interactions. The primary contributions of this work to HRI are: 1) demonstrating a set of studies supporting conceptual replication in HRI; 2) an understanding of how small groups perceive, interact with, and behave with VUIs; 3) a discussion of how VUI social embodiment impact user interaction, behaviors, and perceptions building upon the Human-Robot Group IPO Framework (Sebo et al., 2020); and 4) considerations for future VUI design that promote positive interaction and engagement with VUI agents.

## 2 Related Work

### 2.1 Building Relationships With Voice User Interfaces

Humans are innately social beings. Therefore, when technology engages with people in seemingly social ways, humans respond socially to the device (Nass et al., 1994; Reeves and Nass, 1996). Various features of VUIs have encouraged users to create mental models derived from human-style communication (Kwon et al., 2016). When interacting with another human, we form impressions of the person within the very first minutes of the interaction. In this short amount of time, we decide if the person is safe to interact with and has the potential for a future relationship (Bar et al., 2006; Human et al., 2013). Similar to human-human relationships, users establish impressions of and attribute human characteristics to social agent technologies like VUIs in their very first encounter, even if they only interact with the technology for a few minutes (Dautenhahn, 2004; Powers and Kiesler, 2006; Höflich and El Bayed, 2015; Paetzel et al., 2020). Paetzel et al., 2020 found that competence, perceived anthropomorphism, and likability of robot systems could be established in the first 2 minutes of an interaction and maintained across multiple sessions over time, highlighting the importance of first impressions of social technology. This also suggests that studying first impressions offers a lens to forecasting users’ long-term perception of VUIs.

As humans naturally use affective cues such as facial expressions, vocal prosody, body posture, and social cues like direction of gaze and feedback gestures, it is important to consider how VUIs might better engage users through similar cues to convey personality and intent, and foster natural interactions. The social cues used by agents can be categorized into a taxonomy based on interpersonal communication theory (Feine et al., 2019), mapping agent-human interaction to human-human interaction in terms of kinesics (visually perceivable body movement and gestures), visual, verbal, and auditory (Feine et al., 2019). Voice assistants without embodiment such as Apple’s Siri, Microsoft’s Cortana, and Amazon’s Alexa (without the Echo embodiment) are categorized as having verbal and auditory cues. Siri and Cortana are accompanied with some form of graphics, such as a wavy line or ring on a screen, that react to user’s voice pitch to signal attentiveness. Amazon Echo, a smart speaker, is placed in between these devices with its tower body offering physical presence but without kinesic cues. When Alexa is embodied through Amazon Echo, it uses a light ring to visualize its attention toward the direction of the speaker. These cues act to provide feedback to the user of the device’s active and attentive states, creating a transparent interaction between the user and device (Fong et al., 2003).

The personality design of the VUI agent’s persona can impact user experience as people’s perceptions of devices are often influenced by human-like personality traits (Breazeal, 2004). Personality is a key feature in VUIs and, as such, is an essential factor in promoting user-product relationships and socialization with these devices (Payr, 2013). Agents can embody personality traits through both verbal and nonverbal cues (Tapus et al., 2007) such as light rings in VUI design that indicate attention and allow VUIs to appear engaged. Previous literature also describes that users are more likely to interact with and form a relationship with a device that has a more compelling personality (Kiesler and Goetz, 2002; Breazeal, 2004). Common personalities used in devices such as social robots include tool-like personalities that are dependable and reliable, pet or creature personalities similar to cats or dogs personalities, cartoon personalities with exaggerated traits, artificial being personalities with more mechanistic characteristics, and human-like personalities (Fong et al., 2003).

Whether it is conveyed through affective cues, social cues, nonverbal cues, form, or embodiment, the personality of an agent can help establish relationships with users that contributes to users personifying VUIs as human-like and promoting human-like treatment (Nass and Moon, 2000; Purington et al., 2017). Nass and Moon (2000) described how users tend toward treating computer agents socially and were more accepting of machine-generated personalities that are similar to themselves. Purington et al. (2017) also revealed how Amazon Echo users personified their devices by referring to it by the name Alexa and using personal pronouns for the agent. Their study suggested that personification of the device contributes to user satisfaction based on their observation that personification of Alexa increased levels of user satisfaction. This trend may have developed over time as these home devices are becoming more popular. Moreover, agent personalities and functions lead to users assigning roles to the agents such as “virtual/digital assistant” or “virtual/digital butler” (Payr, 2013) as they are seen to support real time task completion and exert agency on the users behalf. While they are seen less as a companion, these systems still seek to mimic human-human relationships and build trust between the user and the agent (Heuwinkel, 2013).

Users’ socio-engagement often directs technology use and how users develop sustained engagement with the technology. Kidd and Breazeal (2005) highlight three factors which influence long-term relationships with intelligent systems: engagement, trust, and motivation. Increasing engagement relies on the ability to repeatedly draw people into interactions. To help support trust of a system, VUI agents must communicate their capabilities clearly to ensure user expectations are met and appear reliable and credible. Lastly, a system must evoke motivation for users to interact with it, such as being useful or entertaining. Research has investigated factors which impact users’ sustained engagement with technology including social cues, embodiment, and co-presence.

Factors such as co-presence and embodiment impact how users perceive and interact with VUIs (Kidd and Breazeal, 2005; Tapus et al., 2007; Bainbridge et al., 2008). Embodied interaction provides form, substance, and meaning within humans’ physical and social world (Tapus et al., 2007). Since VUI agents can be embodied in numerous ways (for example, a cylindrical smart speaker, a physically animate robot, an interactive display such as on a smartphone, etc.), their diverse physical forms and multi-modal cues allow them to present multiple dimensions of their personality and social cues during interactions. For instance, a personified agent can use verbal and nonverbal cues to appear engaged or disengaged (Tapus et al., 2007). Researchers have found that social presence is greater in physically embodied agents, such as robots, than virtual agents (Heerink et al., 2010; Shinozawa et al., 2002). For example, users were found to provide more respect toward the physically present robot, such as giving it personal space (Bainbridge et al., 2008). Kidd and Breazeal (2005) expanded upon these findings to find that people tend to regard a physically embodied robot more engaging than an animated character. Wainer et al. (2007) postulated “a robot’s physical presence augments its ability to generate rich communication” (Wainer et al., 2007; Deng et al., 2019), emphasizing that physical embodiment provides more natural and social cues that can be utilized to communicate intentions and internal states (Lohan et al., 2010; Deng et al., 2019). Similar results where people find physical co-present robots to be more engaging than digitally embodied forms have been replicated in other labs (Li, 2015). These results suggests a VUI agent’s embodiment can affect users’ engagement and perception, making it a critical feature to consider in VUI agent development. In our work, we explore social embodiment as a combination of the agent’s physical embodiment, interpersonal cues, and social profile (e.g., the name its referred to as). This exploration acknowledges that social embodiment is interconnected with the agent’s social presence (Deng et al., 2019; Segura et al., 2012; Jung and Lee, 2004) and seeks to understand how various VUIs and their social embodiment affect small group interactions.

### 2.2 Robot Interactions in Small Groups

As VUIs become more prominent in social settings and interact with small groups (2–6 people), HRI researchers need to consider how various design features can influence people’s perceptions of VUIs and how they engage with VUIs. Robots and small groups have been studied in various contexts including museum exhibits (Shiomi et al., 2007; Yamazaki et al., 2012; Skantze et al., 2015; Skantze, 2017), shopping malls (Shiomi et al., 2010; Sabelli and Kanda, 2016), day care centers (Tanaka et al., 2007), and other educational settings (Matsuzoe et al., 2014; Hood et al., 2015). Researchers have also studied families as small groups, investigating family members’ utterances to other family members and robots (Short et al., 2017) and non-family intergenerational groups’ interactions with various robots (Joshi and Šabanović, 2019). Our research builds on existing research investigating families as small groups in HRI by exploring families’ interactions in a multi-agent context with different types of VUIs engaging with families.

Families are an interesting small group to study in the HRI community that seeks to understand how robots influence group interactions as current understandings are limited (Hinds et al., 2004; Sebo et al., 2020). There are several factors involved in understanding and analyzing robots in small groups including the characteristics of robots, robot behavior in groups, interaction context, and human-human interactions (Sebo et al., 2020; Ostrowski et al., 2021). Robots’ nonverbal and verbal behaviors can also influence group interactions, leveraging social cues to achieve desired responses or shape group dynamics (Sebo et al., 2020) (more on this above in Section 2.1). There is more growing evidence that human-human interactions are influenced by robots (Sebo et al., 2020), including with families (Ostrowski et al., 2021). While these effects have been studied in the context of robots, it is unclear if these effects can be generalized to VUIs that may not have the same level of physical embodiment.

The Human-robot Group input-process-output (IPO) Framework has been proposed to study group interaction with robots (Sebo et al., 2020) that can be applied to family interactions with VUIs. The framework is drawn from an influential IPO group interaction framework (original: McGrath (1964); adapted by Hackman and Morris (1975) and Sebo et al., 2020). The inputs to the framework are 1) human individual-level factors (i.e., skills, attitudes, personality characteristics), 2) robot individual-level factors (i.e., robot verbal and nonverbal behavior, role of the robot, robot appearance and capabilities), 3) group-level factors (i.e., group type, group composition and size), and 4) environment-level factors (i.e., setting, task characteristics). The processes of the framework are the group-interaction processes, or human-robot interactions and human-human interactions. The outputs for the framework include 1) performance outcomes (i.e., task performance, quality) and 2) other outcomes (i.e., perceptions of the group, perceptions of the robot, attitude change, member satisfaction). Overall, the framework assumes that the “input factors affect performance outcomes through the interaction process” (Hackman and Morris, 1975, p. 6). In our work, we explore multiple aspects of the framework for VUIs, including social robots. For inputs, we leverage differences in VUIs verbal and nonverbal behavior, VUI appearance and capabilities, and group composition and sizes (robot individual-level factors and group-level factors). The VUI based inputs further emphasize our focus on social embodiment. We study human-robot interactions and human-human interactions for group-interaction processes. For outputs, we explore the groups’ perceptions of the robots and member satisfaction.

### 2.3 Replicability in Human-Robot Interaction

In recent years, conversations around replicability in HRI have grown as researchers increasingly seek to understand if there is a replication crisis in HRI (Irfan et al., 2018; Belhassein et al., 2019; Hoffman and Zhao, 2020; Leichtmann and Nitsch, 2020b; Ullman et al., 2021). It is critical for the HRI community to address this area as replication crisis can create fundamental problems of trust that can “cast serious doubt on previous research and undermine the public’s trust in research studies in general” (Hoffman and Zhao, 2020). While replication issues have been well documented in fields such as psychology, medicine, and neuroscience, very few works in HRI have systematically examined if the same replication issues exist in HRI (Irfan et al., 2018; Hoffman and Zhao, 2020). HRI is rampant with low sample sizes resulting in low statistical power, lack of systematic methodological training, and lack of theory around proxemics studies, suggesting that replicability is a key issue to be addressed by the HRI community (Hoffman and Zhao, 2020; Leichtmann and Nitsch, 2020a; Leichtmann and Nitsch, 2020b; Ullman et al., 2021). It is rare to see replications in HRI largely due to the fact that a wide variety of robots are used and those robots are often proprietary, limited, and/or expensive (Baxter et al., 2016; Ullman et al., 2021). Due to these limitations, HRI researchers must “replicate findings across multiple different robots to achieve urgently needed generalizability” (Ullman et al., 2021). Placing too much trust in single studies can lead to detection errors that could be detected in replicated studies (Ullman et al., 2021). When replicating studies, HRI researchers can choose to directly replication a study or conceptually replicate the study (Ward and Kemp, 2019; Ullman et al., 2021). These replication studies can reproduce previous findings as well as reveal novel insights; both of which are valuable outcomes from replication studies (Ullman et al., 2021). In our work, the three studies are not direct replications, however, we follow a similar goal as Leichtmann and Nitsch (2020b) replicating the basic idea and format of the study through a conceptual replication (Ward and Kemp, 2019): A speed dating interaction with small groups engage with three VUIs of various social embodiments followed by three activities.

## 3 Methods

Through a set of three elicitation studies, we explore family interactions with three commercially available VUIs with varying levels of social embodiment. We elected to study family interactions as these devices are mainly marketed for the home. Commercial VUIs are built for the home and the groups that occupy these homes are families, yet, research in studying family group interactions around these technologies is very limited. It is natural that we study how family members interact around these devices and how one’s perception of the agent is influenced by other family members. Previous works have demonstrated that people’s interactions with a robot can impact how people perceive the system (Ljungblad et al., 2012; Sauppé and Mutlu, 2015) and have identified that more research is required to understand how robots shape dynamics of groups and how robot behavior can impact how humans interact with one another in small groups (Jung et al., 2017). Additionally, our previous analysis demonstrated that there are higher amounts of reciprocal behavior, building off of social exchange behaviors, with more socially embodied agents that suggest that group members are influenced by other group members in their interactions and perhaps their perception of the agent (Ostrowski et al., 2021).

### 3.1 Three Exploration Study Design

The first study was exploratory in nature. The focus of this study was to examine how agent personality impacts user perceptions and interactions with three domestic VUIs. Families (*N* = 11 families, 29 participants) interacted with an Amazon Echo (wake word = “Alexa”), a Google Home (“Hey Google”), and a Jibo social robot (“Hey Jibo”). The hypotheses of the first study was that (S1H1) people would engage in more interactions with the agent as the VUI becomes more socially embodied; (S1H2) people would perceive the personalities of the VUIs differently depending on the VUI’s social embodiment; and (S1H3) perceived trust, companionship, competence, and emotional engagement would vary depending on the VUI’s social embodiment. Study 1’s results found that perceived personality is a powerful factor affecting participants’ engagement and perceptions toward VUIs, which raises an ethical concern that certain personality design of an agent could become particularly manipulative towards users, such as hiding/revealing the brand behind the technology. Therefore, to further investigate the influence of branding and how this feature might influence users’ perception, we designed Study 2.

For the second study, to examine the effect of branding on participants’ perception, families (*N* = 11 families, 30 participants) interacted with an Amazon Echo (wake word = “Amazon”), a Google Home (“Hey Google”), and a Jibo social robot (“Hey Jibo”) with researchers priming participants with the agent’s company affiliation and changing the agent’s wake words to reflect their company branding. To emphasize the branding of each agent, experimenters in the study introduced the agents with their company descriptions. The description captures information including “Google is an American multinational technology company that specializes in Internet-related services and products,”^1^ “Amazon, is an American electronic commerce and cloud computing company based in Seattle, Washington”^2^, and “Jibo manufactures and sells electronic robots. The company’s electronic robot lets users talk to Jibo.”^3^ The hypotheses of the second study were that (S2H1) the branding priming would effect how people interacted with the agents; and (S2H2) how people perceived the personalities of the VUIs and perceived trust, companionship, competence, and emotional engagement of the VUIs would be affected by the branding.

The focus of the third study is to specifically explore how interpersonal movement affects participants’ interactions with domestic VUIs, and the factors that influence user’s preference and perception of trust, emotional appeal and engagement, and perceived competence with each agent. Families (*N* = 12 families, 33 participants) interacted with an Amazon Echo Show (wake word = “Computer”), an Amazon Echo Spot (“Alexa”), and a Jibo social robot (“Hey Jibo”). All agents had a touchscreen display that showed various information as the users interacted with them. In addition, the Amazon Echo Spot was modified with a rotating flag that would speed up its rotation speed when a participant called its wake word, adding a mechanical motion. The Amazon Echo Show had no movement within the spectrum, and the Jibo social robot had socially embodied movement. To further help mitigate the effect of participants’ different experience level with VUIs, only those who didn’t own a VUI device were recruited (with the exception of a smartphone) in Study 3. The three agents in Study 3 were selected because they shared a number of important attributes such as a touch screen, having a persona with personality attributes, being able to communicate using far-field speech, having a natural prosodic voice, and the ability to respond to a large number of similar questions that could also help mitigate novelty. Overall, they were situated along a spectrum of social embodiment, bridging between high social embodiment (i.e., Jibo) and low social embodiment (i.e., Computer). The hypotheses of the third study were the same as those from the first study: (S3H1) people would engage in more interactions with the agent as the VUI becomes more socially embodied; (S3H2) people would perceive the personalities of the VUIs differently depending on the VUIs social embodiment; and (S3H3) perceived trust, companionship, competence, and emotional engagement would vary depending on the VUIs’ social embodiment.

### 3.2 Voice User Interface Agents

**FIGURE 1:**
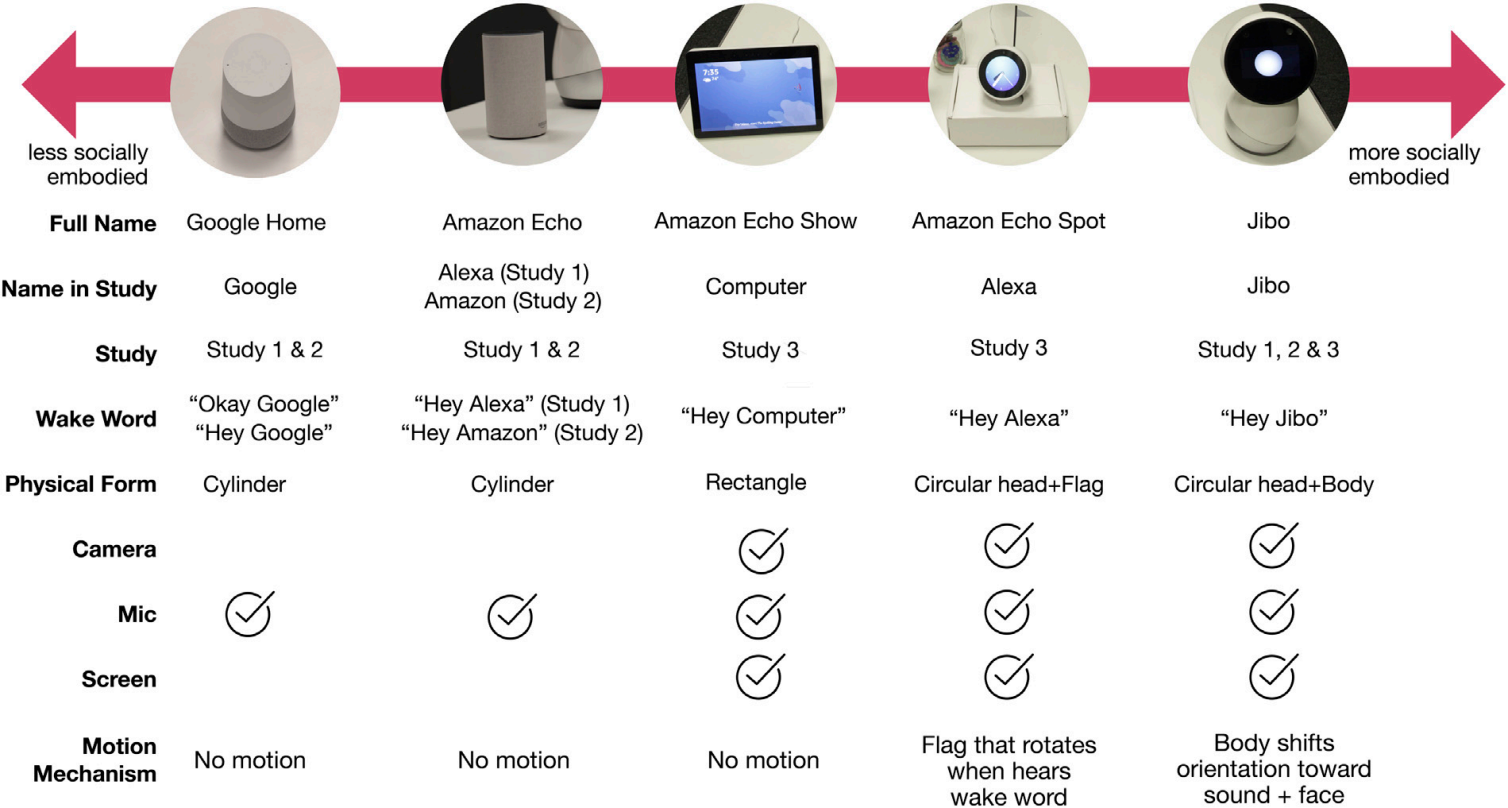
Spectrum of VUIs that represents five commercially available VUIs mapped on to varying levels of embodiment going from less socially embodied to more socially embodied.

We used five commercial VUI devices across the three experiments (Figure 1): Jibo (Study 1–3), Google Home (Study 1, 2), Amazon Echo (Study 1, 2), Amazon Echo Show (Study 3), and Amazon Echo Spot (Study 3).

Jibo is an 11-inch tall and 6-inch wide table-top VUI with a touchscreen face and three degree-of-freedom expressive body that provides contingent motion during an interaction, such as orienting its face and body toward the user upon being called and when a face is detected in its range of view. It also makes small swiveling movements as it speaks. This VUI’s wake word is “Hey Jibo.”

Google Home is a 5.6-inch tall and 3.79-wide cylindrical smart speaker using a virtual agent called Google Assistant to provide the user experience. Users initiate interaction with the wake word “Okay Google” or “Hey Google.”

The Amazon Echo is a 5.8-inch and 3.5-wide cylindrical smart speaker, comparable in height to Google Home. The virtual agency provided through the device is called Alexa, and its wake word is “Hey Alexa” (or “Hey Amazon” for Study 2).

The Amazon Echo Spot is a 3.8-inch tall and 4.1-inch diameter device with a round screen and the Alexa agent. The Amazon Echo Spot was extended to have a motorized fag that continuously rotates above its screen and increases rotation speed when the participants spoke the activation word, “Hey Alexa,” to signal attention through a less socially embodied, mechanical movement. It was designed to appear as an integrated unit with the rotating flag as an additional interaction cue.

The Amazon Echo Show is a 7.4-inch tall and 7.4-inch wide device with a rectangular screen and the Alexa agent. We set its wake word to “Hey Computer,” and it has no motion mechanism.

### 3.3 Participants

Across the three studies, 34 families (92 participants, ages 4–69) from various sociodemographic backgrounds engaged in interactions with VUIs, interviews, and reflective activities. There was only one family (one child and one adult) that overlapped across two studies (Study 1 and Study 3; separated by 1 year).

#### 3.3.1 Study 1

Participants were between 4 and 63 years of age (female = 52%, age M = 23.67, SD = 19.05). Sixteen children (55.2%) and 13 adults (44.8%) participated across the 11 families. Eight participants came from lower-income brackets, 11 participants came from higher income brackets, and 10 declined to answer. Children were all enrolled in school and adults had a minimum of a college associates degree with most having graduate degrees. Families were recruited through emails to the local community and word-of-mouth. All families except for one had children. All participants volunteered to participate, completing an IRB approved consent form. No incentives were offered.

Some participants had been exposed to speaker-based VUIs before. Five families owned an Amazon Echo, one owned a Google Home, and one owned Jibo. One family owned both Amazon Echo and Google Home. Of the VUI device owners, 38% self-reported that they regularly use their devices. Additionally, eight adults reported owning a mobile-platform VUI such as iPhone Siri and 63% of those adults reported using it regularly. Fourteen participants (52%) had never used any type of VUI agents.

#### 3.3.2 Study 2

Participants were between 6 and 63 years of age (female = 58.06%, age M = 21.06, SD = 17.56), including 19 children (59%) and 13 adults (41%). 13 participants came from lower income brackets, seven participants came from higher income brackets, and three declined to answer. All participants volunteered to participate and signed an IRB-approved consent form. No incentives were offered.

Among the 32 participants, three participants owned an Amazon Echo, and one owned a Google Home. 17 participants reported owning a mobile-platform VUI such as Siri, Samsung Bixby, or Google assistant. 15 (47%) had never used any types of VUI agents.

#### 3.3.3 Study 3

Participants were between 6 and 56 years of age (female = 69.26%, age M = 24.42, SD = 17.70), including 17 children (female = 41.18%, age M = 9, SD = 3.16) and 16 adults (female = 37.50%, age M = 39.85, SD = 10.72). Of the 12 families, seven families were one parent and one child; three were one parent and two children; one was four adults and two children; and one was two parents and two children. Five of the 33 participants came from lower income brackets and 28 of the 33 participants came from higher income brackets. Families were recruited through emails to the local community and word-of-mouth. All participants volunteered to participate and signed an IRB approved consent form. No incentives were offered.

For this study, we recruited small groups not owning an Amazon Alexa device, Google Home, or Jibo. Most participants had not interacted with the mentioned VUIs (one adult had interacted with Google Home before the study). Eight people owned an iPhone with the Siri voice agent, and one person owned a smartphone with the Google Assistant voice agent. Others did not acknowledge having voice agents on their smartphones. Therefore, we can view this study as our participants’ first encounter with embodied VUIs, unbiased by previous ownership of a VUI.

### 3.4 Activity Procedures

Across the three studies, all the procedures were structured as elicitation studies with the goal of understanding the reciprocal behaviors of participants when interacting with the VUIs. Elicitation studies were initially proposed as a participatory design methodology to understand users’ preferences for specific interactive situations, such as gestures or symbolic input (Wobbrock et al., 2005; Vatavu and Wobbrock, 2016). We’ve adapted this methodology to our study to understand users’ reciprocal behavior when interacting with VUIs. Since we cannot control for individual features of the commercialized products, such as appearance, size, degree of freedom, voice, or persona of the agent, we structured this study as an elicitation study to maximize the “guessability” of user interactions with VUIs (i.e., understanding how users will interact with VUIs). Additionally, we chose an elicitation study format comparing the three agents side-by-side as elicitation studies focusing on investigating first impressions and exploring people’s interactions and perceptions. By studying how people repeatedly engage with VUIs in a small group, multi-agent format, the elicitation study format allowed us to understand how people engage with these devices and the design features that promote a more natural VUI interaction. The elicitation study in this work is formatted as an adapted “speed dating” (Zimmerman and Forlizzi, 2017). The study mimics human-human speed-dating where participants have the opportunity to interact with multiple other people for short and quick amounts of time. In this work, participants engage in speed-dating with multiple VUIs. This study format was selected over users interacting with one agent at a time because we wanted to compare which agent users prefer to interact within various candidate scenarios and also what information family members exchange while making this decision. This setting closely mimics a real-world situation, such as a family shopping for robots or voice agents in a retail store with multiple options available for purchase. Overall, the elicitation study format enables us to explore interactions with the VUIs holistically as a sum of their features, comparing the complete VUIs side-by-side without strictly comparing one feature at a time.

We designed three activities to examine how people interact with each type of VUI agent and their resulting user perceptions. In the first Agent Exploration Activity (Activity 1), the VUI agents were placed on a table in front of the family members (Figure 3). The order of the agents was randomized for each family. Participants completed an action sheet with 24 directives that each agent could answer. The actions were divided into three categories: information, entertainment, and interpersonal tasks. Information tasks included asking about the weather, news, and general questions or facts. Entertainment tasks focused on pleasure or amusement (i.e., jokes, dancing, music, etc.). Interpersonal tasks focused on learning about the agent’s personality and encouraged deeper engagement with the agent, such as learning about its “thoughts” or “opinions.”

**FIGURE 2:**
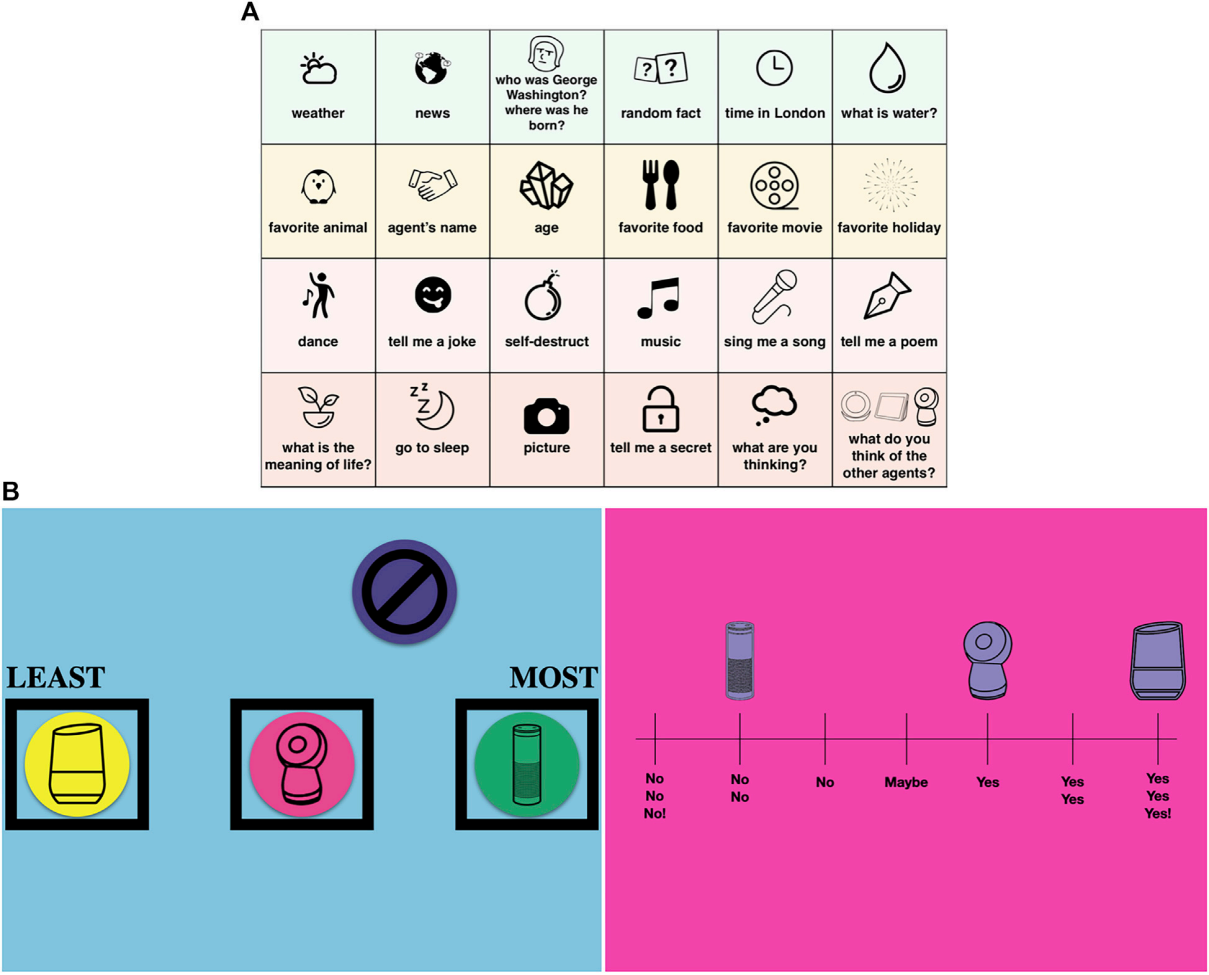
**(A)** Agent action sheet for Activity 1. **(B)** User research tools for Activity 2 and 3.

As a family group, participants completed all the actions on the sheet, having the ability to choose to which agent they asked the question (Figure 2). After all the actions were completed, they engaged in free play with the agents, exploring within and beyond the actions that were presented in the action sheet. Examples of some of these interactions can be found in the external link provided^4^. After free play, participants were asked a series of follow-up questions regarding their thoughts of the three agents.

The next two activities were structured as interactive questionnaires with tactile tools for participants to express their opinions and increase engagement with participants in comparison to a traditional worksheet. The second activity delved into how participants perceived the personalities of the three devices (Activity 2). On a 7-point likert scale from “strongly disagree” (phased as “no, no, no” to be child-friendly) to “strongly agree” (“yes, yes, yes”), participants used three stickers, each shaped like each agent, to express their agreement to a set of statements regarding the agents’ personalities. The personality statements were adopted from Gosling et al. (2003)’s Ten-Item Personality Measure (TIPI), drawn from the Big Five personality test, and adapted for agent personalities (Figure 2).

The third activity was a User Experience Ranking Activity (Activity 3) selected to increase engagement with participants given their age range in comparison to a traditional worksheet (Figure 2). This activity focused on understanding participants’ experience with the agents across several aspects of user interaction. We provided participants with four chips, one with a different graphic for each VUI agent, to indicate their level of agreement (most to least) for each user-experience related statement. If they believed none of the agents applied, a “none” chip was used to indicate their response. The colors of the agent chips were randomized for each participant. The statements comprised 18 categories of user experiences, including agents’ emotional engagement, companionship, competence, and trust. After all the activities, participants were allowed to interact with the agents for as long as they wished.

### 3.5 Data Collection

**FIGURE 3:**
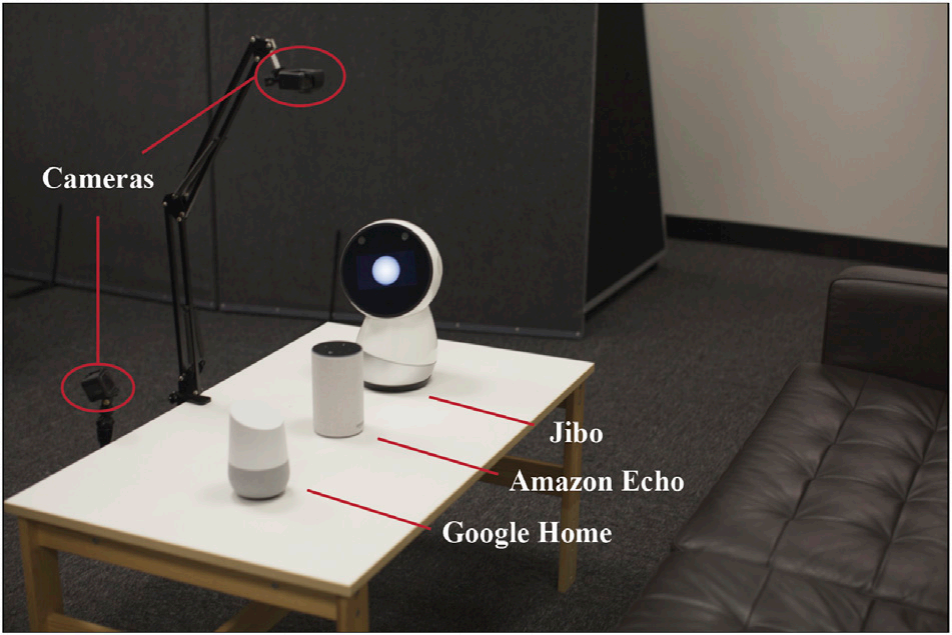
Example study setup with three commercially available at-home VUIs (Jibo, Amazon Echo, and Google Home from Study 1 and 2 shown here) and two cameras for recording the interactions and activities. The same study setup was used for each elicitation study with the varying VUIs.

The sessions were video recorded with a front-face and table-top view (Figure 3). The front-face view recorded interactions and responses; table-top recordings captured participants’ responses to the perceived agent personality and experience ranking activities.

### 3.6 Behavioral Analysis

Our coding scheme was centered on grounding and relational behaviors as previously reported and described in Ostrowski et al. (2021). Grounding behaviors are behaviors like waving, acknowledgement, and relevancy (if a person builds on an agent’s response) while relational behaviors included politeness, positive behaviors, and negative behaviors (Lee et al., 2010). Grounding and relational behaviors were identified from the videos during each interaction episode with an agent (both user trigger and agent response) and the period directly proceeding the interaction as these behaviors could be most linked to a reciprocal interaction following the agent’s actions. On average, participants in the study took 28.74 min to interact with the VUIs and complete the action exploration activity. 1,711 interaction episodes were coded in total, with an average of 142.58 episodes per family and an average length of 7.33 s.

We first reorganized the behaviors into two types of classifications-verbal (e.g., speech, conversation) or nonverbal (e.g., gaze, smile, lean, etc.). Then, we divided the verbal category into three categories-acknowledgment and relevancy, verbal with positive characteristics, and verbal with negative characteristics; and divided the nonverbal category based on what physical part of the body was involved torso, head, hand, eye, and face. In total, 37 different behaviors were coded for in the videos (see Supplementary Material for the full set of behavioral codes). Additionally, all behaviors were coded along two other dimensions: in-sync or mismatched sentiment between the VUI and user; and positive or not positive user sentiment. In-sync and mismatched referred to whether the participant responded to the agent as intended by the agent. For example, if a robot tells a joke, smiling or laughing would be the expected reciprocal behavior. The positive or not positive sentiment (i.e., neutral or negative) referred to how the participant regarded the interaction with the agent. For example, laughing after an agent’s incorrect response was coded as not positive and mismatched. It was interpreted as positive or not by the participant’s behavior and context was accounted for in the coding scheme for each behavior. The second dimension revealed that positive sentiment behaviors were observed significantly more frequently than neutral or negative sentiment. Moreover, we tracked which agent users’ attentions were directed at while themselves or others were interacting with each agent. Two researchers each individually coded 50% of the data and, then, reviewed the others coding. Discrepancies were discussed and resolved.

## 4 Findings

Our results demonstrate how families interact with and perceive agents’ personalities, trust, companionship, competence, and emotional engagement, increasingly as VUIs become more socially embodied. More so, this is consistently demonstrated across the three studies of these agents. Results demonstrate how small groups interact with VUIs and with each other. When comparing across three agents, we used Friedman Chi-Squared test and post-hoc Wilcoxon tests with Holms correction. When comparing between two agents, we used Kruskal-Wallis H-test. We used a non-parametric test since most of our data samples for each study were small (less than 40). Similar results were found with adults and kids, therefore, results for the whole population are reported. After each study’s results, study specific design considerations are highlighted as well.

### 4.1 Study 1: Jibo, Amazon Echo, and Google Home

Study 1 was the initial study conducted to understand in an elicitation study format how, and if, these agents would be interacted with and perceived. The results for this study and the following sections are organized by the interaction analysis, personality measure analysis, and the acceptance measure analysis along the dimensions of trust, companionship, competence, and emotional engagement.

#### 4.1.1 Interaction

**FIGURE 4:**
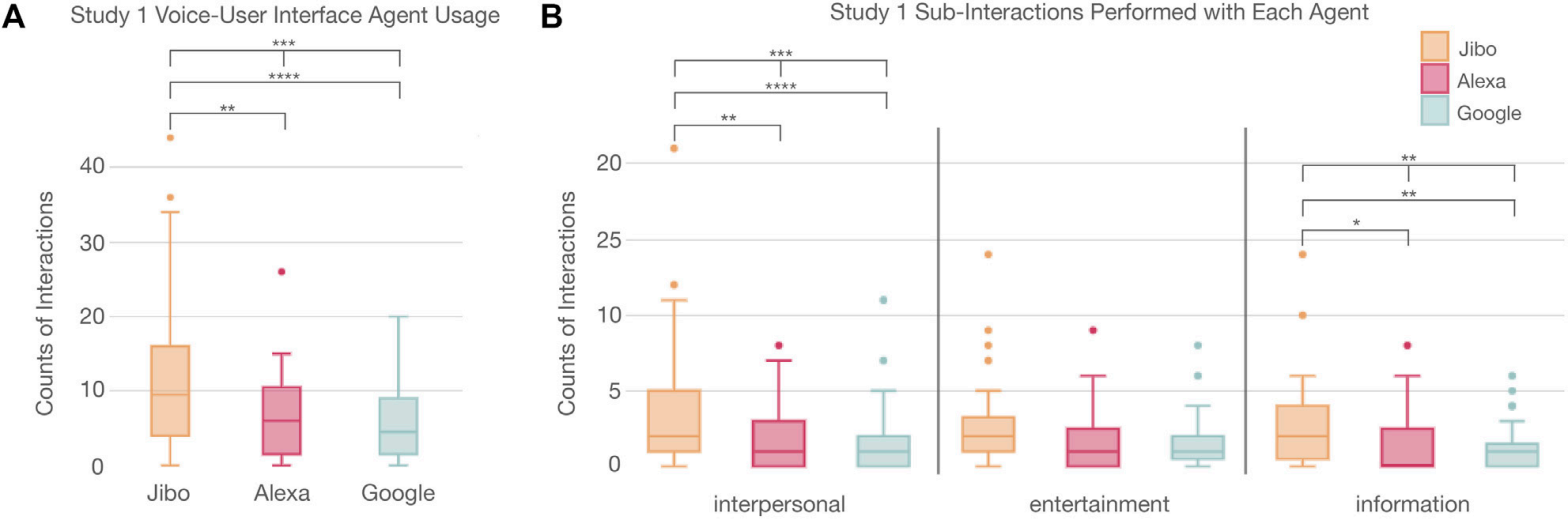
**(A)** Significant trend in usage of voice agents between participants showing Jibo had a significant higher usage than Alexa or Google. **(B)** Distribution of the interactions completed with various agents showing that participants engaged with agents differently for interpersonal and information based interactions.

Participants interacted significantly more with agents that were more socially embodied (*p* < 0.001***) (Figure 4A; Supplementary Table S1). A post-hoc Wilcoxon test with Holm correction revealed that Jibo was interacted with significantly more than Alexa (*p* < 0.01**) and Google (*p* < 0.0001****), respectively. Delving deeper into the interactions, the types of interactions that participants completed with each agent were also analyzed. The actions were divided into three categories: entertainment, information, and interpersonal (Figure 4B), and the actions were compared using a Friedman Chi-Squared test and post-hoc Wilcoxon tests with Holm correction. Overall, participants explored all three types of tasks with each of the VUI agents. We then looked deeper at the interactions to understand the distribution of actions with each agent. For these results, only those who interacted with all three agents were included to ensure all agents were represented per participant to better understand how the actions were distributed. Significant difference across the three agents was found for the interpersonal actions (*p* < 0.001***) and information interactions (*p* < 0.01**). The entertainment actions were not significantly different across agents. A post-hoc test revealed that more interpersonal actions were completed with Jibo than Alexa (*p* < 0.01**) and Google (*p* < 0.0001****), respectively. More information actions were also completed with Jibo than Alexa (*p* < 0.05*) and Google (*p* < 0.01**), respectively.

#### 4.1.2 Personality

**FIGURE 5:**
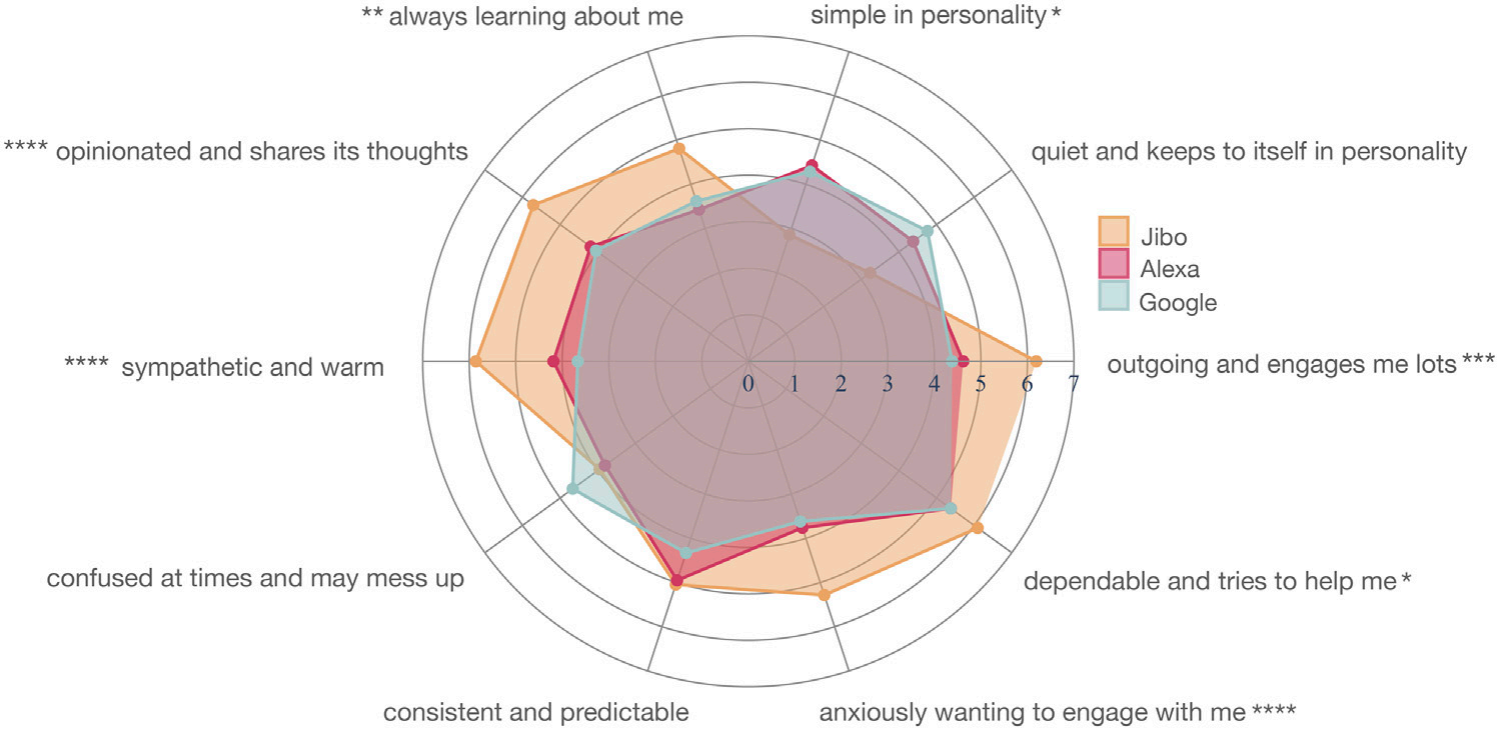
Personality mapping for Jibo (orange), Alexa (red), and Google (blue) in Study 1

The agents were assessed along the Big 5 personality metrics, broken into the foundational 10 traits in this analysis. For Study 1, the three agents’ personalities were perceived differently (Figure 5; Supplementary Table S2). The agents’ personality was significantly different for seven personality traits, including “outgoing and engages me lots” (*p* < 0.001***), “simple in personality” (*p* < 0.05*), “always learning about me” (*p* < 0.01**), “opinionated and shares its thoughts” (*p* < 0.0001****), “sympathetic and warm” (*p* < 0.0001****), “anxiously wanting to engage with me” (*p* < 0.0001****), and “dependable and tries to help me” (*p* < 0.05*). The agents’ personality traits were not perceived as significantly different for “quiet and keeps to itself,” “confused at times and may mess up,” and “consistent and predictable.”

For the significantly different personality traits, a post-hoc Wilcoxon test with Holm correction revealed that the main significant difference was between Jibo and Alexa followed by Jibo and Google. Jibo was perceived as significantly more “outgoing and engages me lots” than Alexa (*p* < 0.001***) and Google (*p* < 0.001***), respectively. Jibo was also perceived significantly less “simple in personality” than Alexa (*p* < 0.01**) and Google (*p* < 0.01**), respectively. Jibo was perceived significantly more as “always learning about me” than Alexa (*p* < 0.01**) and Google (*p* < 0.01**), respectively. For “opinionated and shares its thoughts,” Jibo was perceived higher in this trait than Alexa (*p* < 0.001***) and Google (*p* < 0.01**), respectively. Jibo was perceived as more “sympathetic and warm” than Alexa (*p* < 0.01**) and Google (*p* < 0.001***), respectively. For “anxiously wanting to engage with me,” Jibo was perceived higher in this trait than Alexa (*p* < 0.01**) and Google (*p* < 0.01**), respectively. Lastly, Jibo was perceived as more “dependable and tries to help me” than Alexa (*p* < 0.05*) but not perceived differently from Google. There was no perceived difference in traits between Alexa and Google.

**FIGURE 6:**
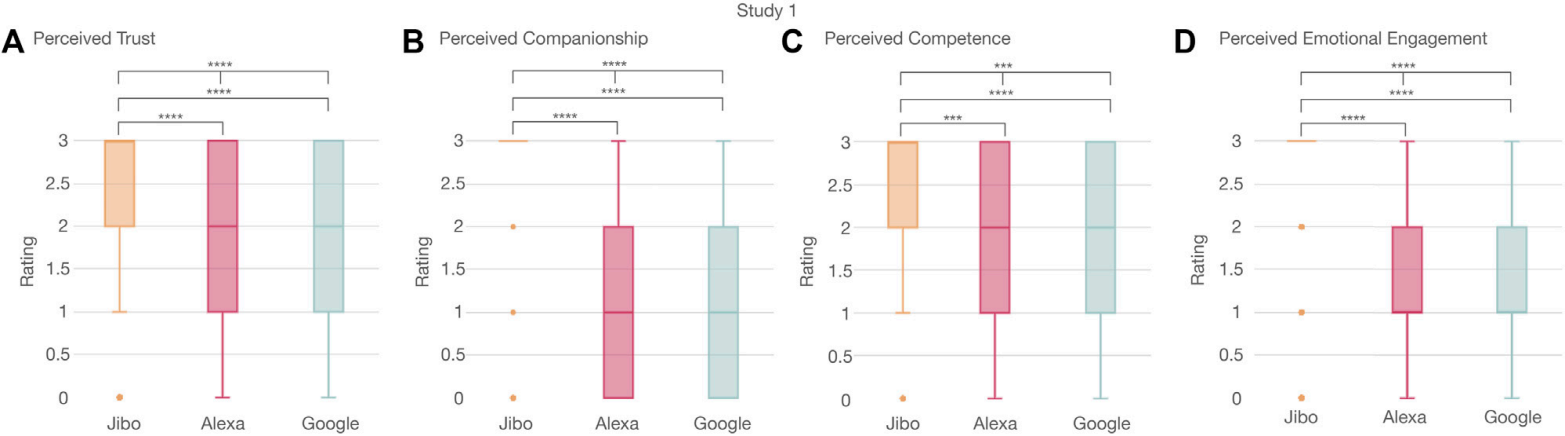
Significant trend in perceived trust, companionship, competence, and emotional engagement of voice agents between participants showing Jibo was significantly perceived as **(A)** more trustworthy, **(B)** more like a companion, **(C)** more competent, and **(D)** more emotionally engaging than Alexa or Google.

The acceptance measurement was analyzed according to trust, companionship, competence, and emotional engagement metrics (full results and statistical tests in Supplementary Table S3). Participants perceived Jibo as more trustworthy followed by Alexa then Google (*p* < 0.0001****) (Figure 6A). A post-hoc Wilcoxon test with Holm correction supported this trend. Jibo was perceived as more trustworthy than Alexa (*p* < 0.0001****) and Google (*p* < 0.0001****), respectively. This trend was also seen with companionship where Jibo was perceived as most like a companion followed by Alexa and then Google (*p* < 0.0001****) (Figure 6B). By a post-hoc Wilcoxon test with Holm correction, Jibo was perceived as greater a companion than Alexa (*p* < 0.0001****) and Google (*p* < 0.0001****), respectively. For perceived competence, Jibo was perceived as more competent than Google or Alexa (*p* < 0.001***) (Figure 6C). A post-hoc test revealed Jibo was perceived as more competent than Alexa (*p* < 0.001***) and Google (*p* < 0.0001****), respectively. Lastly, emotional engagement followed the same trend with Jibo perceived as more emotionally engaged than Alexa or Google (*p* < 0.0001****) (Figure 6D). Again, Jibo was perceived as more emotionally engaging than Alexa (*p* < 0.0001****) and Google (*p* < 0.0001****), respectively.

#### 4.1.3 User Experience

##### 4.1.3.1 Study 1 Design Considerations

The findings from this study offer several design considerations for VUIs. When considering designing VUIs in contexts where families will engage with them, designers must understand how VUIs’ perceived personalities and social embodiments are associated with their perceived roles. When designing to optimize both the “assistant-like” role and “companion-like” role for a device, designers can focus on designing VUI’s social embodiment that incorporates social personalities that engage with users in ways to foster relationship. In this study, Jibo utilized its social embodiment and more complex and compelling personality to increase user engagement. When considering personality, designers can be aware that more social personalities were perceived as more compelling, resulting in increased engagement. Therefore, to support social embodiments in VUIs with compelling personalities, designers can promote warm, outgoing, and thoughtful agent personalities that also express desires and intentions for interacting with users. Overall, designers can promote engagement with agents through expressive agent personality, utilizing both voice, graphics, movement, and social embodiment to communicate with users. However, since the personalities and forms of VUIs are powerful engagement factors influencing user experience with agents in terms of perceived sociability and emotional engagement with the agent, ethical design principles need to be adopted as such VUI’s have the potential to be particularly persuasive.

### 4.2 Study 2: Jibo, Amazon Echo, and Google Home With Branding Emphasis

Study 2 builds upon Study 1 to understand how reinforcing an agent’s branding impacts how these agents are interacted with and perceived. In Study 2, the wake word of the Amazon Echo was changed to “Amazon” to reinforce the brand of the agent. Therefore, the results for the agents are reported as Jibo, Amazon, or Google, respectively. As in the previous sections, the results for Study 2 are organized by the interaction analysis, personality measure analysis, and the acceptance measure analysis along the dimensions of trust, companionship, competence, and emotional engagement. We provide additional analysis in this section comparing Alexa from Study 1 to Amazon in Study 2 that use the same embodiment (Amazon Echo) but with different wake words.

#### 4.2.1 Interaction

**FIGURE 7:**
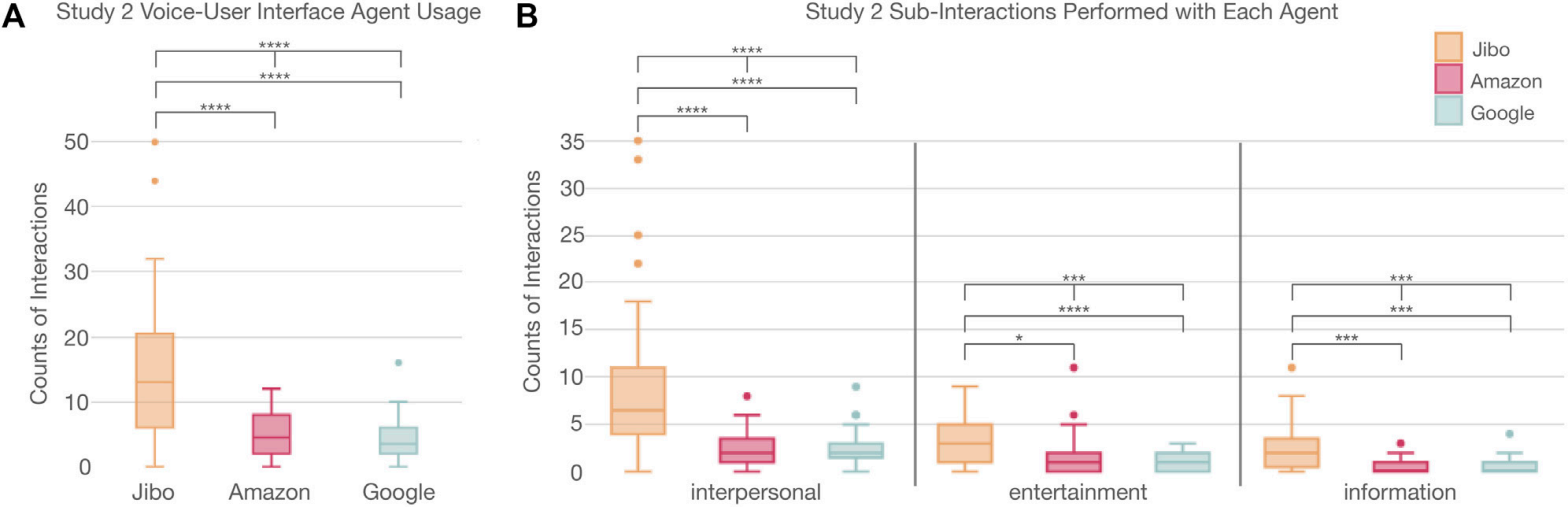
**(A)** Significant trend in usage of voice agents between participants showing Jibo had a significant higher usage than Amazon or Google Home. **(B)** Distribution of the interactions completed with various agents showing that participants engaged with agents different for interpersonal, entertainment, and information based interactions.

A similar interaction trend was found to Study 1 as more socially embodied agents were interacted with more (*p* < 0.0001****) (Figure 7A; Supplementary Table S4). A post-hoc Wilcoxon test with Holm correction revealed participants interacted with Jibo significantly more than Amazon (*p* < 0.0001****) and Google (*p* < 0.0001****), respectively. Overall, there was no significant difference between Alexa in Study 1 and Amazon in Study 2 (Kruskal-Wallis H-test *χ*2 (1, *N* = 59) = 1.21; *p* > 0.05).

The interactions were once again categorized as interpersonal, information, or entertainment actions. For these results, only those who interacted with all three agents were included to ensure all agents were represented per participant to better understand how the actions were distributed. The three agents were significantly different for interpersonal (*p* < 0.0001****), information (*p* < 0.001***), and entertainment actions (*p* < 0.001***) (in contrast, with Study 1, where only the first two action categories were significantly different across the agents) (Figure 7B; Supplementary Table S4). Post-hoc Wilcoxon tests with Holm correction supported the trend observed in Study 1. Significantly more interpersonal actions were completed with Jibo than Amazon (*p* < 0.0001****) and Google (*p* < 0.0001****), respectively. Significantly more entertainment actions were completed with Jibo than Amazon (*p* < 0.05*) and Google (*p* < 0.0001****), respectively. Lastly, participants completed significantly more information actions with Jibo than Amazon (*p* < 0.001***) and Google (*p* < 0.001***), respectively. Alexa in Study 1 was chosen significantly more than Amazon for interpersonal actions (Kruskal-Wallis H-test *χ*2 (1, *N* = 92) = 7.20; *p* < 0.01**). For entertainment and information actions, there were no statistical difference between them though Alexa was chosen slightly more frequently than Amazon (entertainment: Kruskal-Wallis H-test *χ*2 (1, *N* = 48) = 2.31; *p* > 0.05; information: Kruskal-Wallis H-test *χ*2 (1, *N* = 40) = 2.03; *p* > 0.05).

#### 4.2.2 Personality

**FIGURE 8:**
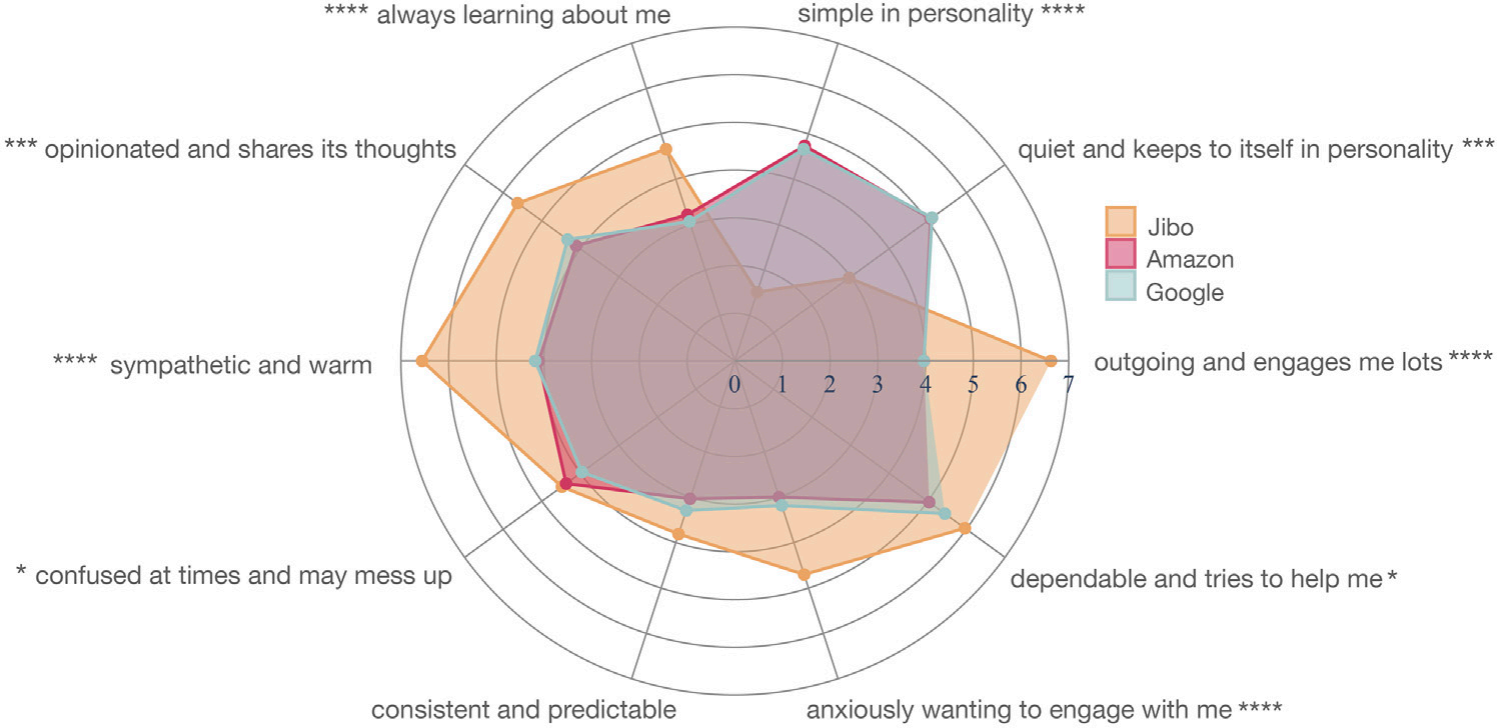
Personality mapping for Jibo (orange), Amazon (red), and Google (blue) in Study 2

When branding was reinforced for the agents, there were nine personality traits (out of 10) that were significantly different across the three agents (Figure 8 and Supplementary Table S5). These included “outgoing and engages me lots” (*p* < 0.0001****), “quiet and keeps to itself” (*p* < 0.001***), “simple in personality” (*p* < 0.0001****), “always learning about me” (*p* < 0.0001****), “opinionated and shares its thoughts” (*p* < 0.001***), “sympathetic and warm” (*p* < 0.0001****), “confused at times and may mess up” (*p* < 0.05*), “anxiously wanting to engage me” (*p* < 0.0001****), and “dependable and tries to help me” (*p* < 0.05*).

For the significantly different personality traits, a post-hoc Wilcoxon test with Holm correction supported this trend for all seven significant personality traits with the main significant difference between Jibo and Amazon followed by Jibo and Google. For “outgoing and engages me lots”, Jibo was perceived as significantly more outgoing than Amazon (*p* < 0.0001****) and Google (*p* < 0.0001****), respectively. Jibo was also perceived as significantly more “quiet and keeps to itself” than Amazon (*p* < 0.001***) and Google (*p* < 0.01**), respectively. Jibo was seen as having significantly less of “simple in personality” than Amazon (*p* < 0.001***) and Google (*p* < 0.0001****), respectively. For “always learning about me”, Jibo was perceived as significantly more this trait than Amazon (*p* < 0.001***) and Google (*p* < 0.001***), respectively. Jibo was also perceived as significantly more “opinionated and shares its thoughts” than Amazon (*p* < 0.01**) and Google (*p* < 0.05*), respectively. For “sympathetic and warm”, Jibo was perceived as more of this trait than Amazon (*p* < 0.0001****) and Google (*p* < 0.0001****), respectively. The post-hoc Wilcoxon test with Holm correction was not significant for pairwise comparison between the agents for “confused at times and may mess up”. For “anxiously wanting to engage with me”, Jibo was perceived as more of this trait than Amazon (*p* < 0.01**) and Google (*p* < 0.01**). Lastly, Jibo was perceived as significantly more “dependable and tries to help me” than Amazon (*p* < 0.05*). Alexa in Study 1 and Amazon were only significantly different for one trait, “consistent and predicatable,” (Kruskal-Wallis H-test *χ*2 (1, *N* = 48) = 9.22; *p* < 0.01**) with Alexa in Study 1 being perceived as more consistent and predictable than Amazon.

#### 4.2.3 User Experience

**FIGURE 9:**
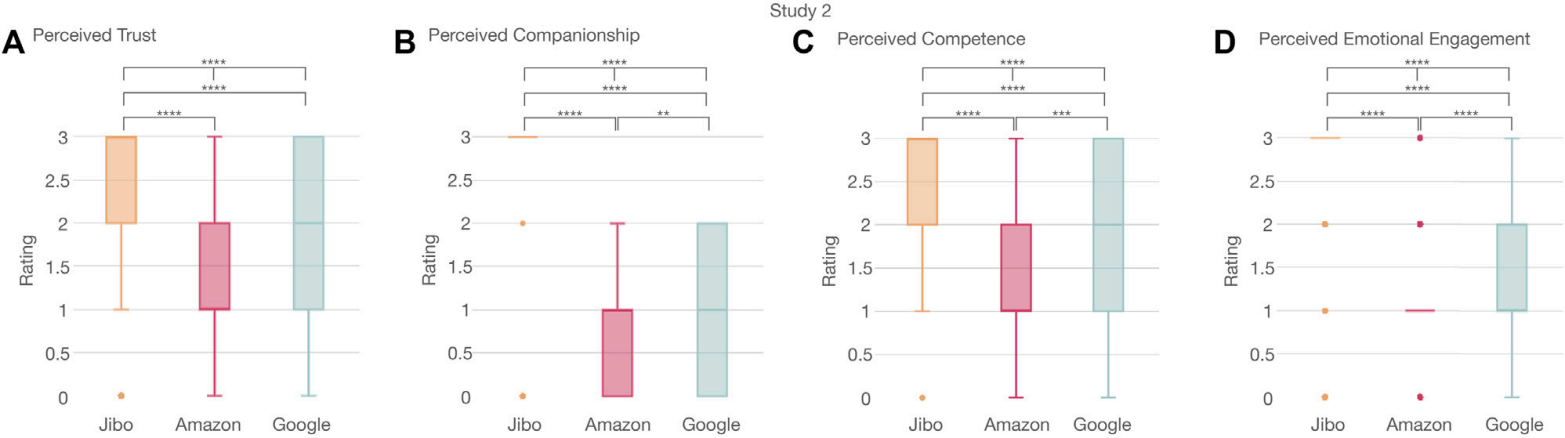
Significant trend in perceived trust, companionship, competence, and emotional engagement of voice agents between participants showing Jibo was significantly perceived as **(A)** more trustworthy, **(B)** more like a companion, **(C)** more competent, and **(D)** more emotionally engaging than Amazon or Google. Google was perceived as **(B)** more like a companion, **(C)** more competent, and **(D)** more emotional engaging than Amazon.

The acceptance measurement was analyzed according to trust, companionship, competence, and emotional engagement metrics. This analysis revealed the most significant difference between the agents resulting from the branding reinforcement as seen between Google and Amazon (full results and statistical tests are shown in Supplementary Table S6). Perceived trust was significantly different between the three agents (*p* < 0.0001****) (Figure 9A). Jibo was perceived as significantly more trustworthy than Amazon (*p* < 0.0001****) and Google (*p* < 0.0001****), respectively. Google was also perceived as more trustworthy than Amazon (*p* < 0.01**). Perceived companionship was also significantly different between the three agents (*p* < 0.0001****) (Figure 9B). Jibo was perceived as significantly more like a companion than (*p* < 0.0001****) and Amazon (*p* < 0.0001****), respectively. Google was also perceived as significantly more like a companion than Amazon (*p* < 0.01**). The three agents were perceived significantly different for competence (*p* < 0.0001****) (Figure 9C). Jibo was perceived as significantly more competent than Google (*p* < 0.0001****) and Amazon (*p* < 0.0001****). Google was also perceived significantly more competent than Amazon (*p* < 0.001***). Lastly, perceived emotional engagement was also significantly different across the three agents (*p* < 0.0001****) (Figure 9D). Jibo was perceived significantly more emotionally engaging than Google (*p* < 0.0001****) and Amazon (*p* < 0.0001****), respectively. Google was also perceived significantly more emotionally engaging than Amazon (*p* < 0.0001****). Alexa in Study 1 was perceived significantly differently from Amazon for trust, competence, and emotional engagement (trust: Kruskal-Wallis H-test *χ*2 (1, *N* = 364) = 10.66; *p* < 0.01**; competence: Kruskal-Wallis H-test *χ*2 (1, *N* = 368) = 17.68; *p* < 0.0001****; emotional engagement: Kruskal-Wallis H-test *χ*2 (1, *N* = 650) = 22.85; *p* < 0.0001****). Though Alexa was perceived more companion like than Amazon, it was not statistically significant (Kruskal-Wallis H-test *χ*2 (1, *N* = 135) = 1.26; *p* > 0.5).

#### 4.2.4 Study 2 Design Considerations

The findings from this study offer design considerations for VUIs around socially embodiment and socially-embodied naming. As in Study 1, the social robot’s high degree of social embodiment promoted increased interaction and more positive perception of the agent. We hypothesized that a name the agent is referred to as (personified vs. company affiliated) may introduce different perceptions around trust, competence, companionship and emotional engagement of the agent. The socially embodied named Alexa was perceived as more trustful, more competent, and more like a companion than the less socially embodied named Amazon. Considerations should be made around how the agent’s name, i.e., wake word, can influence user acceptance. However, ethical considerations should be made around using personified names as it could conceal company branding and deteriorate transparency. Designers should consider how socially embodied personified names can and cannot provide greater transparency into who is creating and designing the technology while also allowing greater user choice into which VUI agents users buy. Another consideration is around how branding effects novelty. Brand knowledge may decrease the time necessary to become familiar with the device and/or reach a stable usage pattern (Leite et al., 2013).

### 4.3 Study 3: Jibo, Amazon Echo Show, and Amazon Echo Spot

Study 3 builds upon the previous two studies to examine the impact of an agent’s movement on participants’ perceptions and interactions with the agent. As in the previous sections, the results for Study 3 are organized by the interaction analysis, personality measure analysis, and the acceptance measure analysis along the dimensions of trust, companionship, competence, and emotional engagement.

#### 4.3.1 Interaction

**FIGURE 10:**
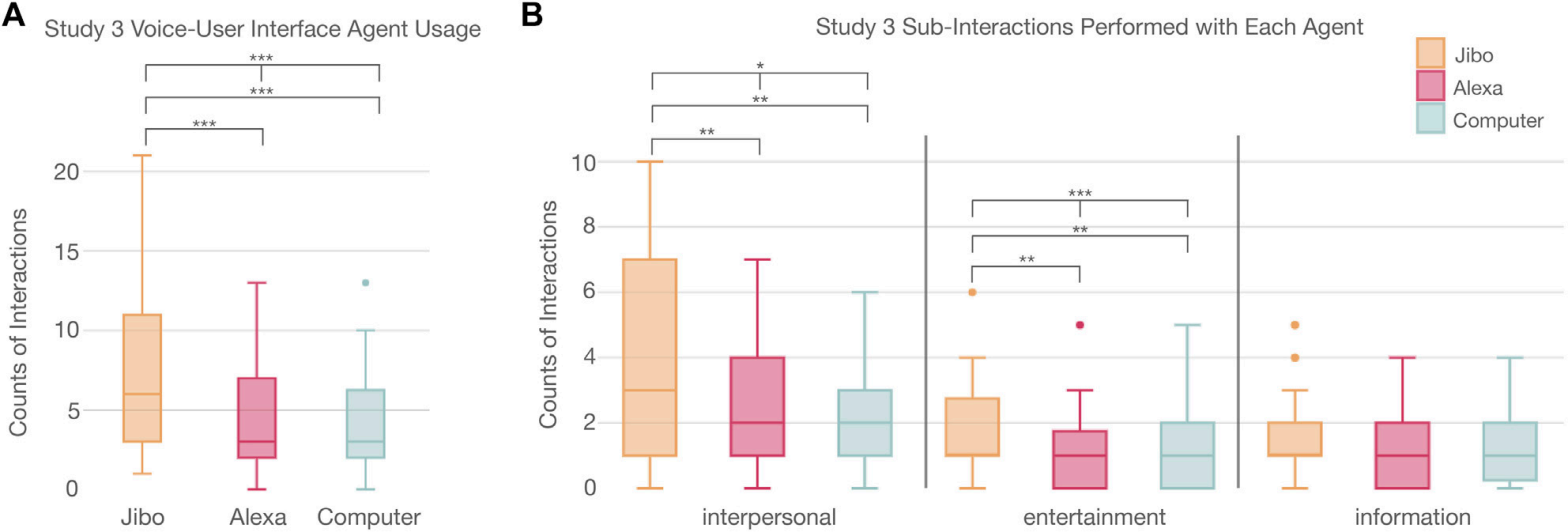
**(A)** Significant trend in usage of voice agents between participants showing Jibo had a significant higher usage than Alexa or Computer. **(B)** Distribution of the interactions completed with various agents showing that participants engaged with agents different for interpersonal and entertainment based interactions.

Similar to the previous two studies, in Study 3, participants interacted significantly more with agents that were more socially embodied (*p* < 0.001***) (Figure 10; Supplementary Table S7). A post-hoc Wilcoxon test with Holm correction supported this trend. Participants interacted significantly more with Jibo (7.36 ± 5.47) than Alexa (*p* < 0.001***) and Computer (*p* < 0.001***), respectively.

Delving deeper into the interactions, the types of interactions that participants completed with each agent were also analyzed. The actions were divided into three categories-entertainment, information, and interpersonal. For these results, only those who interacted with all three agents were included to ensure all agents were represented per participant and get a better understanding of how the actions were distributed. The actions were then compared using a Friedman Chi-Squared test and post-hoc Wilcoxon tests with Holm correction.

Significant difference across the three agents was found for interpersonal (*p* < 0.05*) and entertainment actions (*p* < 0.001***). The information actions were not significantly different across agents. A post-hoc Wilcoxon test with Holm correction supported this trend for interpersonal and entertainment actions. Participants completed significantly more interpersonal actions with Jibo than Alexa (*p* < 0.01**) and Computer (*p* < 0.01**). For entertainment actions, participants completed significantly more of these actions with Jibo than Alexa (*p* < 0.01**) and Computer (*p* < 0.01**), respectively.

#### 4.3.2 Personality

**FIGURE 11:**
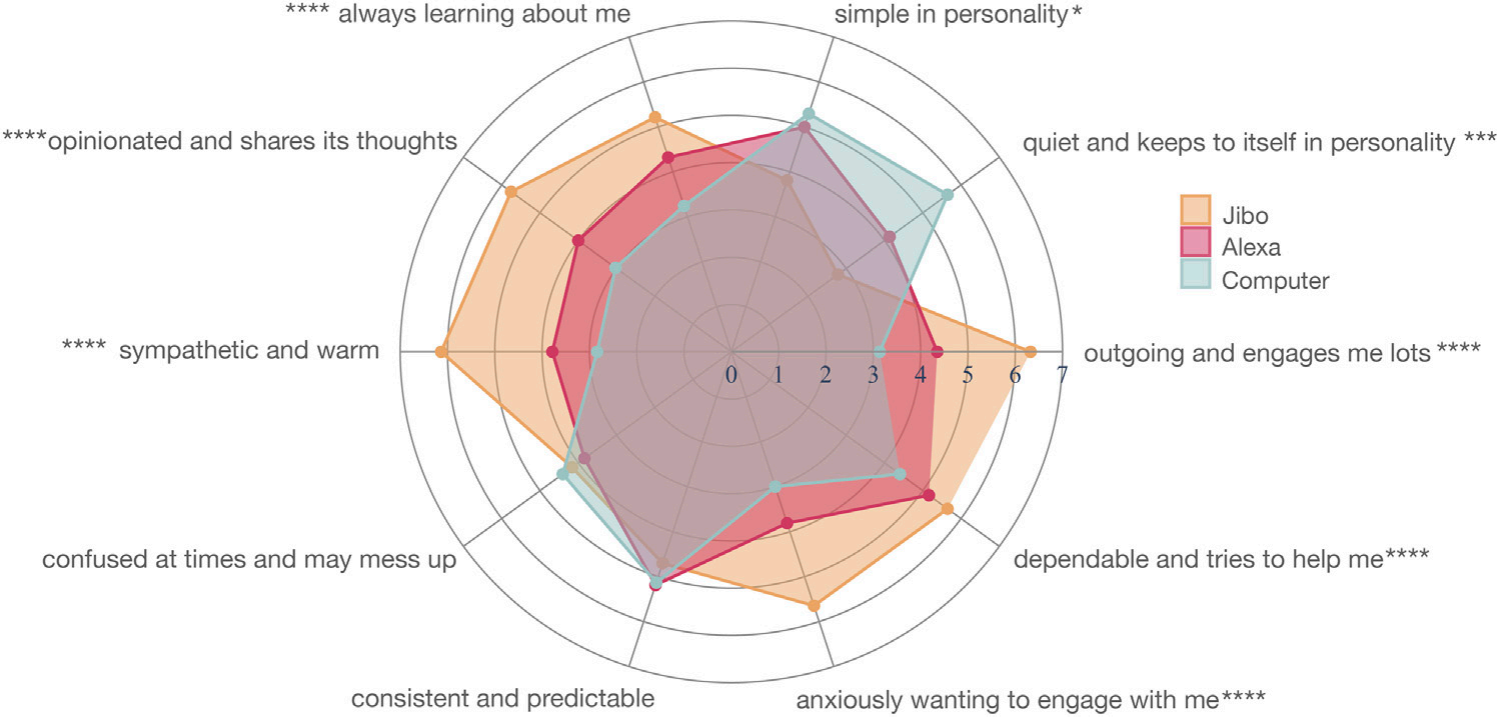
Personality mapping for Jibo (orange), Alexa (red), and Computer (blue) in Study 3

When movements were reinforced for the agents, there were eight personality traits (out of 10) that were significantly different across the three agents (Figure 11; Supplementary Table S8). These included “outgoing and engages me lots” (*p* < 0.0001****), “quiet and keeps to itself” (*p* < 0.001***), “simple in personality” (*p* < 0.05*), “always learning about me” (*p* < 0.0001****), “opinionated and shares its thoughts” (*p* < 0.0001****), “sympathetic and warm” (*p* < 0.0001****), “anxiously wanting to engage me” (*p* < 0.0001****), and “dependable and tries to help me” (*p* < 0.0001****).

For the significantly different personality traits, a post-hoc Wilcoxon test with Holm correction supported this trend for all eight significant personality traits with the main significant difference between Jibo and Alexa followed by Jibo and Computer. Jibo was perceived as significantly more ‘outgoing and engages me lots” than Alexa (*p* < 0.0001****) and Computer (*p* < 0.0001****), respectively. Computer was also perceived significantly more outgoing and engaging than Alexa (*p* < 0.01**). For “quiet and keeps to itself,” Jibo was perceived as significantly less this trait than Alexa (*p* < 0.001***) and Computer (*p* < 0.0001****), respectively. Computer was perceived as significantly more quiet than Alexa (*p* < 0.001***). For “simple in personality,” Jibo was perceived significantly less this trait than Alexa (*p* < 0.001***) and Computer (*p* < 0.05*), respectively. Jibo was perceived significantly more in the “always learning about me” trait than Alexa (*p* < 0.05*) and Computer (*p* < 0.0001****), respectively. Computer was perceived as significantly less in this trait than Alexa (*p* < 0.001***). Jibo was perceived as significantly more “opinionated and shares its thoughts” than Alexa (*p* < 0.0001****) and Computer (*p* < 0.0001****), respectively. Computer was perceived significantly less opinionated than Alexa (*p* < 0.01**). For “sympathetic and warm,” Jibo (6.14 ± 1.34) was perceived as significantly more this trait than Alexa (*p* < 0.0001****) and Computer (*p* < 0.0001****), respectively. Computer was also perceived significantly less “sympathetic and warm” than Alexa (*p* < 0.01**). Jibo was perceived significantly more as “anxiously wanting to engage with me” than Alexa (*p* < 0.0001****) and Computer (*p* < 0.0001****), respectively. Computer was also perceived significantly less this trait than Alexa (*p* < 0.01**). Lastly, Jibo was perceived significantly more “dependable and tries to help me” than Alexa (*p* < 0.05*) and Computer (*p* < 0.01**), respectively. Computer was perceived significantly less dependable than Alexa (*p* < 0.001***).

#### 4.3.3 User Experience

To further investigate how the agent’s movement affected participants’ experience with the agents, the acceptance measurement was grouped into four categories-trust, companionship, competence, and emotional engagement metrics. Overall, there were significant differences between the agents for each of the four categories (full results and statistical tests shown in Supplementary Table S9).

**FIGURE 12:**
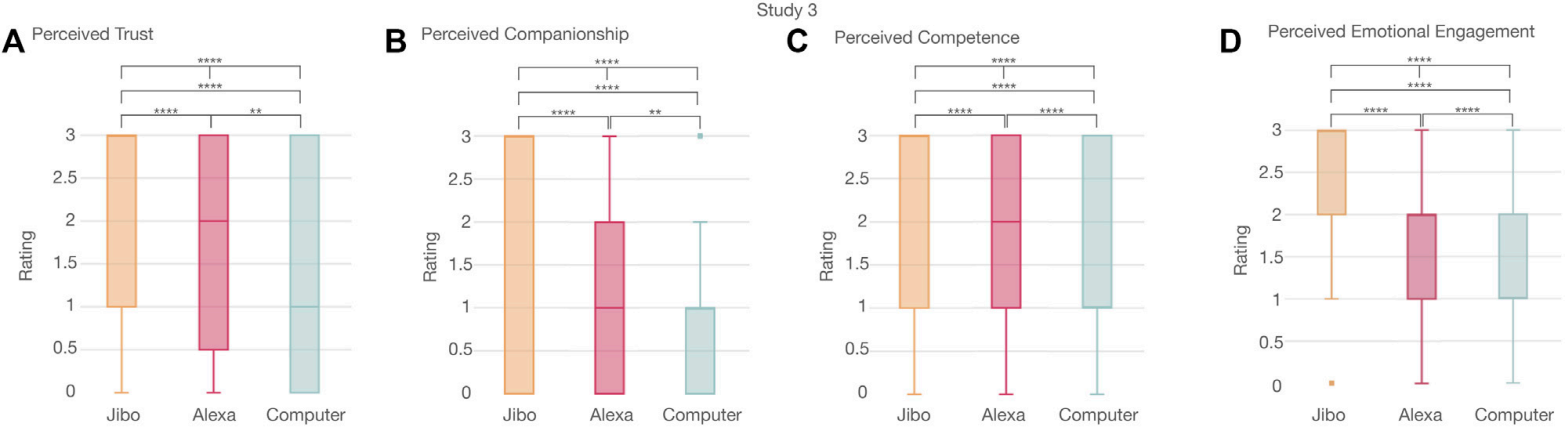
Significant trend in perceived trust, companionship, competence, and emotional engagement of voice agents between participants showing Jibo was significantly perceived as **(A)** more trustworthy, **(B)** more like a companion, **(C)** more competent, and **(D)** more emotionally engaging than Alexa or Computer. Alexa was perceived as **(A)** more trustworthy, **(B)** more like a companion, **(C)** more competent, and (d) more emotionally engaging than Computer.

Perceived trust was significantly different between the three agents (*p* < 0.0001****) (Figure 12A). Jibo was perceived as more trustworthy than Alexa (*p* < 0.0001****) and Computer (*p* < 0.0001****), respectively. Computer was perceived as significantly less trustworthy than Alexa (*p* < 0.01**). Perceived companionship was also significantly different between the three agents (*p* < 0.0001****) (Figure 12B). Jibo was perceived as significantly more like a companion than Computer (*p* < 0.0001****) and Alexa (*p* < 0.0001****), respectively. Computer was perceived as significantly less than a companion than Alexa (*p* < 0.01**). The three agents were perceived significantly different for competence (*p* < 0.0001****) (Figure 12C). Jibo was perceived as significantly more competent than Computer (*p* < 0.0001****) and Alexa (*p* < 0.0001****). Computer was perceived as less competent than Alexa (*p* < 0.0001****). Lastly, perceived emotional engagement was also significantly different across the three agents (*p* < 0.0001****) (Figure 12D). Jibo was perceived as significantly more emotional engaging than Computer (*p* < 0.0001****) and Alexa (*p* < 0.0001****), respectively. Computer was perceived significantly less emotionally engaging than Alexa (*p* < 0.0001****).

#### 4.3.4 Behavioral Analysis

As previously reported in Ostrowski et al. (2021), thirty-seven behaviors that were reciprocal in nature and invoked by the agent’s response to an action request were observed between the agents and group members. The results reported here are from the same population sample as in Ostrowski et al. (2021). Since there was no significant difference between children and adults for behaviors (Mann-Whitney U test; U (1) = 30,253.00; *p* > 0.05), we reported the results as a whole population. To compare between the three agents, we performed Friedman Chi-Squared test and post-hoc Wilcoxon tests with Holms correction. These tests were selected as our sample size is less than 30 requiring a non-parametic test. In Table 1, the tests comparing the three agents for behaviors are shown, including overall and individual behavior results. The statistically significant results suggest that participants engaged in more reciprocal behaviors with the social robot Jibo rather than Alexa or Computer. Participants also engaged in more human communications such as smiling, laughing, and complimenting with Jibo compared to the other agents. This is consistent with other data measures reported in this paper and provides a deeper lens into not only how frequently participants interacted with the agents but the types of behaviors the agents’ interactions invoked.

**TABLE 1:** Comparing reciprocal behaviors observed around the three VUI agents that vary in the degree of social embodiment. The overall result across the combined 37 coded behaviors and the seven behaviors that showed statistically significant difference between the agents are highlighted.

Individual behavior	Behavior description	Jibo mean ± std	Alexa mean ± std	Computer mean ± std	Friedman test	Post-hoc wilcoxon with holm correction
Total	Total number of occurrences of 37 coded behaviors	28.79 ± 18.89	12.22 ± 7.96	11.21 ± 6.52	*χ*2 (2, *N* = 1711) = 19.22	Jibo vs. Alexa: Z = 4.75, *p* < 0.01**
					*p* < 1e-04****	Jibo vs. Comp: Z = 4.91, *p* < 0.001***
Agent luring attention	Agent’s behavior promotes group member to direct attention and body language to the agent	1.62 ± 1.19	0.23 ± 0.44	0.0 ± 0.0	*χ*2 (2, *N* = 24) = 19.95	Jibo vs. Alexa: Z = 2.87, *p* < 0.01**
					*p* < 1e-04****	Jibo vs. Comp: Z = 3.06, *p* < 0.01**
Relevancy	Group member builds on an agent’s response	2.1 ± 1.25	0.55 ± 0.99	0.75 ± 0.85	*χ*2 (2, *N* = 68) = 24.99	Jibo vs. Alexa: Z = 3.60, *p* < 0.001***
					*p* < 1e-04****	Jibo vs. Comp: Z = 3.44, *p* < 0.001***
Smiling	Group member smiles due to agent’s action	4.87 ± 3.53	1.18 ± 1.38	1.29 ± 1.23	*χ*2 (2, *N* = 241) = 38.66	Jibo vs. Alexa: Z = 4.77, *p* < 1e-04****
					*p* < 1e-04****	Jibo vs. Comp: Z = 4.55, *p* < 1e-04****
Physical imitation	Group member imitates agent’s response using their body	1.25 ± 0.5	0.0 ± 0.0	0.0 ± 0.0	*χ*2 (2, *N* = 5) = 8.00	N/A due to small number of samples
					*p* < 0.05*	
Looking away	Group member breaks eye-contact with the agent to look elsewhere	2.74 ± 1.89	1.48 ± 1.55	1.26 ± 1.06	*χ*2 (2, *N* = 148) = 14.02	Jibo vs. Alexa: Z = 2.76, *p* < 0.01**
					*p* < 0.001***	Jibo vs. Comp: Z = 3.33, *p* < 0.001***
Laughing	Group member laughs at the agent’s response	3.86 ± 3.00	1.21 ± 2.10	1.0 ± 1.09	*χ*2 (2, *N* = 170) = 28.72	Jibo vs. Alexa: Z = 3.83, *p* < 0.001***
					*p* < 1e-04****	Jibo vs. Comp: Z = 4.25, *p* < 1e-04****
Complimenting	Group member gives a compliment to the agent	1.27 ± 0.79	0.09 ± 0.30	0.18 ± 0.60	*χ*2 (2, *N* = 17) = 11.64	Jibo vs. Alexa: Z = 2.50, *p* < 0.05*
					*p* < 0.01**	Jibo vs. Comp: Z = 2.04, *p* < 0.05*

In addition to individual behaviors, the agents’ interactions also invoked human-human behaviors, or behaviors between group members. In Table 2, the tests comparing the three agents for group behaviors are shown, including overall and group behavior results. Delving into the various types of group behaviors, we highlight glancing at others, private conversations, and defending behaviors. As with the individual behaviors, the group behavior analysis suggests that participants engaged in more group behaviors with the social robot Jibo compared with the other agents. Jibo was the only agent that participants defended to other group members. Again, this group behavior analysis supports that VUIs can promote social interactions among group members as well as with the VUI itself.

**TABLE 2:** Comparing group behaviors observed around the three VUI agents that vary in the degree of social embodiment. The overall result across the combined behaviors and subsequent three behaviors showed statistically significant difference between the agents.

Group behavior	Behavior description	Jibo mean±std	Alexa mean±std	Computer mean±std	Friedman test	Post-hoc wilcoxon with holm correction
Total	Total number of occurrences of coded group behaviors	7.72 ± 6.34	2.91 ± 2.12	2.75 ± 2.91	*χ*2 (2, *N* = 428) = 27.28	Jibo vs. Alexa: Z = 4.35, *p* < 0.01****
					*p* < 1e-04****	Jibo vs. Comp: Z = 4.44, *p* < 0.001***
Defending an agent	A group member defended an agent’s action or response to another group member	1.50 ± 1.00	0.0 ± 0.0	0.0 ± 0.0	*χ*2 (2, *N* = 4) = 8.0	N/A due to small number of samples
					*p* < 0.05*	
Private conversations	Group member turning to another group member to whisper and/or have a private conversation about agent and/or agent’s response	2.7 ± 2.1	1.09 ± 1.12	0.87 ± 1.14	*χ*2 (2, *N* = 23) = 14.80	Jibo vs. Alexa: Z = 3.13, *p* < 0.01**
					*p* < 0.001***	Jibo vs. Comp: Z = 3.49, *p* < 0.001***
Glancing at others	Group member glances at another group member	5.77 ± 4.74	2.19 ± 1.58	2.19 ± 2.46	*χ*2 (2, *N* = 31) = 22.94	Jibo vs. Alexa: Z = 4.20, *p* < 1e-04****
					*p* < 1e-04****	Jibo vs. Comp: Z = 4.06, *p* < 1e-04****

#### 4.3.5 Study 3 Design Considerations

The findings from this study support that form and animacy can be used as a method to promote expressiveness and convey non-verbal social cues to foster engagement with the users. Social cues, nonverbal, gestures, movement, and expressiveness can indicate attentiveness and interest to the user, allowing users to personify the VUI’s reactions and emotions that can strengthen users’ relationships with the technology. Through leveraging socially contingent movements and verbal and nonverbal social cues such as orientating gaze and conveying emotions, designers can promote human-agent interactions and human-human interactions in small group contexts, including families as shown in this study. These findings and design considerations can be expanded beyond families to other small group contexts including promoting collaborative teamwork and joint action. By incorporating socially contingent movements and social cues into VUIs, designers can also promote behaviors that foster reciprocal likability between users and the technology.

## 5 Discussion

Our work studies how small groups interact with and perceive VUIs in multi-user, multi-agent environments through three elicitation studies that focus on VUIs as holistic units of study and provides a model of conceptually replicating HRI speed dating interactions with VUIs and small groups. In our discussion below, we reflect upon how VUI sociability mechanisms and their social embodiment impact user interaction, behaviors, and perceptions of VUI trust, companionship, competence and emotional engagement. We highlight how our findings build upon the Human-Robot Group IPO Framework (Sebo et al., 2020), how our replication study format revealed novel insights, and considerations for future VUI design.

### 5.1 Mechanisms of Sociability

Sebo et al.’s (2020) IPO framework emphasizes that embodiment through robot behavior and robot movements are crucial input influencers affecting the group interaction process and perceptions of the robots. Consistent with the framework, current results from our studies found that participants engaged with and perceived the VUI agent as more engaging when it has interpersonal movement and higher social embodiment. We found that interpersonal actions, in particular, were the most popular across all agent types, emphasizing that users are drawn to interact socially and interpersonally with VUI agents (Nass and Moon, 2000; Bickmore and Picard, 2005). Participants interacted with a VUI with the highest social embodiment, i.e., social robot Jibo, the most overall, and also completed the most social interactions with it, suggesting that the robot’s interpersonal movement and social embodiment drew users to interact with it (Kiesler and Goetz, 2002; Breazeal, 2004).

Interestingly, while participants interacted with agents similarly for information actions, participants perceived each VUI’s competency differently. Information actions showed no significant usage difference among agents, suggesting that all agents provided sufficient transactional information to participants. However, participants viewed Jibo’s competency to be significantly higher than other agents. This result suggested that interpersonal movement and social embodiment may enhance the perceptions of competency (Cassell, 2000; Bickmore and Cassell, 2005). In particular, Jibo scored the highest on measures that would require the agent to learn more about participants (e.g., to be adaptive to the user), or to exhibit greater autonomy or agency (such as being proactive). Jibo’s interpersonal movement and social-emotional cues conveys more attention and intentions (Fong et al., 2003), allowing users to perceive Jibo as more competent than other VUI agents.

The five agents across three studies have varying levels of social embodiment, including their form factor, movement, personality, and the design of the interactions being more transactional or more relational (Payr, 2013). For example, Jibo as a social robot had the highest degree of social embodiment including its attentive social-emotional cues and interpersonal delivery of interactions. Google Home, Amazon Echo, Amazon Echo Show, and Amazon Echo Spot were more focused on their transactional functionalities that opt for simpler designs with fewer social-emotional cues. Cho et al., 2019 emphasizes that users expect to have emotional exchanges and build relationships with VUIs. Our study results also show that users engaged with VUIs through affective, social cues and trust, and more so with an agent with higher social embodiment. Such engagement may lead users to form an emotional bond with the agent (Forlizzi and Battarbee, 2004). Bickmore and Cassell (2005) emphasize the importance of mimicking face-to-face conversations and interactions with embodied conversational agents. Non-verbal social cues such as facial expressions, head movement and posture shifts can all impact a perceptions of collaborativeness and cooperativeness of agents (Cassell, 2000; Breazeal, 2004). In contrast to the other VUI agents with mechanical movement (Amazon Echo Spot with a flag) or no movement (Amazon Echo, Amazon Echo Show, and Google Home), Jibo engages users through a repertoire of interpersonal movements and socio-emotive cues such as turning to look at people when they wave or say “Hey Jibo,” using expressive motions to convey emotional sentiments during speech, and using postural shifts at natural pauses in speech. These emotively expressive and attentive cues could have encouraged participants to form a stronger and deeper emotional bond with Jibo even during first encounters, promoting more interpersonal interactions, companionship, and emotional engagement (Forlizzi and Battarbee, 2004).

Specifically, in Study 3, two of the VUIs had a combination of movement (Jibo had socially mediated movement and Alexa had a mechanical flag) and attention indicating features (Jibo had gaze and movement and Alexa had a mechanical flag) that may have promoted reciprocal behaviors from the participants. While we designed Alexa with a rotating flag as an additional interaction cue that could signal attentiveness and promote reciprocal behaviors, the result showed that Alexa and Computer were not significantly different from one another with regards to reciprocal behaviors. This finding may suggest that a repetitive, less socially embodied movement is no more beneficial than a light ring indicator that both Alexa and Computer possessed.

### 5.2 Voice User Interface Perception and Small Group Interaction

Social-emotional cues and embodiment, along with interpersonal movements, draw users into interacting with VUIs and enhancing user perceptions of personalities, trust, competence, companionship and emotional engagement (Cassell, 2000; Forlizzi and Battarbee, 2004). Consistent through the three studies, across all categories of user experience, Jibo with higher levels of social embodiment was rated significantly higher than the other VUI agents that had lower levels of social embodiment (Amazon Echo, Amazon Echo Show, Amazon Echo Spot, and Google Home). Deeper analysis revealed that participants perceived Jibo’s personality as the most outgoing, sympathetic, and wanting to share thoughts and engage with the users. The robot’s social embodiment could have promoted trust and credibility in its engagement with users by enhancing transparency to understand the robot’s internal state (Kidd and Breazeal, 2005; Tapus et al., 2007; Cho et al., 2019). While Jibo’s personality was perceived as most outgoing, sympathetic, and wanting to share thoughts and engage with users in all studies, there were some differences between Alexa’s and Computer’s perceived personalities in Study 3. In this study iteration, Alexa (with the mechanical rotating flag) and Computer were perceived differently in terms of their personalities, even though it was the same agent (Alexa) and only the embodiment and wake word were different. Combined the different embodiments and names created varying levels of social embodiment with Alexa having a higher social embodiment than Computer. Alexa was perceived significantly more outgoing, engaging, inquisitive about the user, and opinionated than Computer. Computer was perceived significantly quieter and into itself. Similar trends were seen with perceived trust, competence, companionship, and emotional engagement with Alexa perceived higher than Computer. These results further suggest that embodiment has an effect on trust and credibility (Kidd and Breazeal, 2005; Tapus et al., 2007).

The presented studies focus on a single exposure, whereas it is fair to wonder if the findings would persist after multiple encounters. However, recent studies have demonstrated that first impressions of a system are lasting in future interactions (Paetzel et al., 2020) and usage patterns are consistent over the first few days of interacting with an agent as users explore and form a relationship with the device (Bentley et al., 2018; Singh, 2018). These prior works highlight that there are differences between VUI agents with varying social embodiments (stationary smart speakers versus social robots) in the first day of interaction, and that these usage patterns remain consistent for weeks. Both short- and long-term studies with VUI agents (e.g., smart speakers, smart displays, social robots) are valuable to the HRI community, especially given that these agents still represent a newer, less explored technology within small groups. Studies with VUI agents can also look at how different generations interact with such devices, as this work has done.

Small group, multi-user scenarios have been of great interest to the HRI community (Jung et al., 2017; Sebo et al., 2020). In this study, we focused the small group interactions around families. In their review paper on robots in groups and teams, Sebo et al., 2020 distinguished three different types of groups when interacting with robots-intimacy groups, task groups, and loose associations (Lickel et al., 2000; Sebo et al., 2020). Intimacy groups refer to close personal relationship groups like friends, romantic partners, or families. Task groups are characterized as teams that are oriented around a shared task or interest. Loose associations represent both temporary groups of people, like people who are waiting in line at a bank, and longer-term shared interests groups like neighbors (Clark and Mils, 1993; Lickel et al., 2000). Sebo et al., 2020 emphasizes that the majority of the HRI research centers on loose association and task groups (Sebo et al., 2020), while our studies focus on family groups investigating the interactions between intimacy groups and robots. As the IPO framework emphasizes, the verbal and nonverbal behaviors of robots can shape participants’ perceptions and interactions with the devices (Sebo et al., 2020). Results from our current in-lab mixed-agent experiments support the framework and found that participants are more encouraged to interact and engage emotionally with VUI agents when they are designed with higher social embodiment. By measuring how families perceive and interact with three VUI agents in a multi-agent environments, we found that agents with more engaging, personable, and “friend-like” personalities and higher social embodiment can foster deeper social connections with the users.

### 5.3 Replicability

The studies presented in this paper represent an iterative study processes that promotes conceptual replication. Lack of replication is a critical area to address in HRI studies (Irfan et al., 2018; Hoffman and Zhao, 2020; Leichtmann and Nitsch, 2020b; Ullman et al., 2021). Through conceptual replication, this paper demonstrates how consistent the results are for VUIs with higher social embodiment. In all three studies, the social robot was more highly interacted with, perceived as having a more warm, outgoing, and complex personality, and perceived as more trustworthy, competent, companion-like, and emotionally engaged. These are key sociability aspects that can impact how successful interactions are with users, collaboration, and cooperativeness in small groups (Cassell, 2000; Breazeal, 2004). The small group multi-agent speed dating format presented in this study enabled multiple rounds of replication to further support this consistent trend. These studies demonstrate how conceptual replication can be increasingly incorporated into HRI to promote generalizability (Ullman et al., 2021), reveal novel insights for future exploration (Ullman et al., 2021), and increase the community’s trust in HRI research studies Hoffman and Zhao (2020).

### 5.4 Voice User Interface Design Considerations

The speed-dating elicitation format used in this paper allows designers and researchers to explore VUI agents in a holistic manner across a spectrum of various design features (i.e., embodiment, branding, etc.) and different contexts (i.e., small group vs. individual, multi-agent, generational). This format is especially valuable for working with commercialized products where we are unable to control features such as appearance, degree of freedom, voice, or agent persona. Overall, the format provides a new way to investigate user perception and interaction with VUI agents to inform the next design iterations of VUIs. While studying how people interact and perceive VUIs, it is critical to consider how these systems can and should be designed ethically as technologies, such as VUIs, have the potential to be a powerful persuasive tool. Further work must seek to understand how VUI design features impact users in not only small group, multi-agent contexts but potentially persuasive contexts as well.

### 5.5 Considerations and Future Directions

In understanding how small groups interact with VUIs, it is important to treat VUIs as holistic devices. By treating the device as a culmination of all its features and not just a sum of each, researchers can more fully explore how users interact with them (Cambre and Kulkarni, 2019). First impressions are also critical time points to study as lasting impressions of likeability and overall feelings towards VUIs are most likely to occur in the first few minutes interaction with a system (Paetzel et al., 2020). When designing our study, we ensured that participants could complete each action with any of the agents and did not include tasks that only one or two of the agents could complete. This prevented participants from gaining perspectives that one agent was superior over the others because it did more functions than the others. While this was not a focus in our study, future studies could explore how small groups engage with VUIS that have varying levels of utility. The three studies were iteratively developed and conceptually replicated the previous studies. Future work could directly replicate each of the three studies and conceptually replicate the study in a real-world setting outside of the lab. Related to our statistical analysis, we did not account for a group effect of participants’ behaviors influencing one another. Future work could investigate the participants’ behaviors further using a mixed-effects model. The last study consideration to be mindful of is that these results may not apply to cultures outside of the United States (Katagiri et al., 2001).

Our results demonstrate that VUI design features around social embodiment such as verbal and nonverbal social cues and social mechanisms may impact how small groups engage with and perceive VUIs. Future studies could explore how specific design features elicit interaction and which design features are suitable for varying types of interaction. In addition, trust and companionship can be further studied to understand how these relational components developed in user’s first impressions are maintained depending on varying design features of VUIs. While our study only looked at first impressions, it is important for future work to understand how VUI embodiment and social presence impacts long-term engagement and how these design features impact group dynamics.

## 6 Conclusion

In this study, families as small groups interact with three commerical VUIs in an elicitation study with a speed dating format to understand how small groups and individuals interact with and perceive VUIs. By studying VUIs in this format, we investigated VUIs as a holistic unit that can be compared with other VUIs. The three studies described in this paper demonstrate the consistency of the results, addressing conceptual replication to better understand results. In each study, VUIs with higher levels of social embodiment were perceived as more trustworthy, competent, companion-like, and emotionally engaged. These VUIs also had the largest amount of interaction. Branding, embodiment, and agent wake word greatly influenced how lesser socially embodied agents were interacted with and perceived. The discussion in this paper focused on how these results interact with mechanisms of sociability and the Human-Robot Group IPO Framework. This paper concludes with a discussion around how this work contributes to supporting replicability in HRI and considerations for how VUIs should be designed.

## Data Availability

The datasets presented in this article are not readily available because, we will have a future publication that makes the dataset available. Requests to access the datasets should be directed to AO, akostrow@media.mit.edu.

## Footnotes

1
https://en.wikipedia.org/wiki/Google.

2
https://en.wikipedia.org/wiki/Amazon_(company).

3 Adapted from https://www.bloomberg.com/profile/company/1232907D:US.

4
https://youtu.be/g0cfP_T1Ld0.
