# Supplementary material for: Speed Dating with Voice User Interfaces: Understanding How Families Interact and Perceive Voice User Interfaces in a Group Setting
Source: Front Robot AI. 2022 Jan 14;8:730992. doi: 10.3389/frobt.2021.730992 (PMC8819708; doi:10.3389/frobt.2021.730992)
Supplement: Supplementary file 1 [file DataSheet2.PDF]

Reciprocal Behaviors

Acknowledgement

1. Short Response - replying to an agent after a response with: “Yeah” “Ok” “Alright”
2. Mhm - replying with thoughtful sound like “mhmm”
3. Answering Question
4. Ask a Follow-up Question
5. Exclamation - “wow!” “amazing!” “ooh” “aww” etc.
6. Relevancy - built upon the robot’s previous utterance with sentence

Body Physical

7. Move Toward Agent
8. Move Away from Agent
9. Touch Agent
10. Point at Agent - includes gesturing towards agent
11. Shrug
12. Luring - being pulled away from one agent to another
13. Nudging another person

Head Physical

14. Nod - up/down
15. Shake Head - left/right
16. Tilt Head - listening or intrigued
17. Imitation - imitate agent’s movement physically
18. Imitation Dance: imitating robot’s dance moves
19. Generic Dance: dancing to a speaker, tapping out rhythm

Hand Physical

20. Waving - greeting
21. Hand Gesture - palm or hand turned up

Eye

22. Switch Eye Contact - look up and down a lot from agent
23. Look Away - cut off eye contact from agent
24. Glancing at others

Face

25. Smile

26. Judgemental Eyebrow Movement - “really”
27. Inquisitive Eyebrow Movement
28. Downward Frown - physical acknowledgment
29. Mouth Open/Agape

Positive Verbal

30. Talking among each other - private conversations and inaudible side comments (also purple)
31. Laugh
32. Thank you
33. Compliment
34. Defend - advocate on behalf of agent from another person’s critique

Negative Verbal

35. Insult - “I don’t like Computer” etc.
36. Judgemental Verbal - disappointed with agent’s answer, phrases like “Um.. okay then”, “That wasn’t really what I asked” etc.
37. Angry Verbal - negative verbal catch-all if no other fit
38. Interrupt - cut off the robot mid-reply, often to stop agent

Additional Metrics

Verbal: Behaviors 1-6 and 29-37

Behaviors in purple are human-human behaviors

Behaviors above in red are not considered for the following labels:

Positive

- A participant’s behavior was assessed situationally and determined positive

Not Positive

- Assessed situationally - includes neutral and negative emotions

In Sync

- If the participant and agent both display Positive/Not Positive sentiment
- Ex. Jibo tells a joke, participant expresses glee and laughs (P and P)

Mismatch

- If the participant and agent are not in agreement in terms of Positive/Not Positive
- Ex. Computer tells a joke, participant stares back blankly (P vs. NP)
