# Supplementary material for: Speed Dating with Voice User Interfaces: Understanding How Families Interact and Perceive Voice User Interfaces in a Group Setting
Source: Front Robot AI. 2022 Jan 14;8:730992. doi: 10.3389/frobt.2021.730992 (PMC8819708; doi:10.3389/frobt.2021.730992)
Supplement: Supplementary file 2 [file DataSheet1.PDF]

Supplementary Material

1 STUDY 1 DETAILED RESULTS

**Interaction Type**	**Jibo** mean±std	**Alexa** mean±std	**Google** mean±std	**Friedman test**	**Post-hoc Wilcoxon with Holm correction**
Total Interaction	12.90±11.10	6.71 ± 6.00	6.07 ± 5.41	χ^2(2, N=84)=17.18 p<.001***	Jibo vs Alexa: Z=3.29, p<.01**<br>Jibo vs Google: Z=4.13, p<1e-04****
Interpersonal	8.68 ± 5.76	4.32 ± 2.87	4.05 ± 3.08	χ^2(2, N=66)=18.10 p<.001***	Jibo vs Alexa: Z=3.13, p<.01**<br>Jibo vs Google: Z=3.75, p<1e-04****
Entertainment	3.27 ± 3.48	2.32 ± 2.23	1.95 ± 1.96	χ^2(2, N=66)=2.00 p>.05	N/A
Information	3.36 ± 3.40	1.77 ± 2.39	1.50 ± 1.79	χ^2(2, N=66)=10.34 p<.01**	Jibo vs Alexa: Z=2.22, p<.05*<br>Jibo vs Google: Z=2.69, p<.01**

Table S1. Comparing interaction types around the three VUI agents in Study 1. The overall, interpersonal, and information interactions showed statistically significant difference between the agents are highlighted.

**Personality Trait**	**Jibo** mean±std	**Alexa** mean±std	**Google** mean±std	**Friedman test**	**Post-hoc Wilcoxon with Holm correction**
Simple in personality	2.86 ± 2.46	4.43 ± 1.78	4.29 ± 1.59	χ^2(2, N=63)=7.48, p<.05*	Jibo vs Alexa: Z=2.62, p<.01**<br>Jibo vs Google: Z=2.60, p<.01**
Quiet & keeps to itself in personality	3.24 ± 2.23	4.38 ± 1.63	4.76 ± 1.76	χ^2(2, N=63)=2.35, p>.05	N/A
Outgoing & engages me lots	6.19 ± 1.03	4.62 ± 1.24	4.38 ± 1.36	χ^2(2, N=63)=16.48, p<.001***	Jibo vs Alexa: Z=3.45, p<.001***<br>Jibo vs Google: Z=3.47, p<.001***
Dependable & tries to help me	6.10 ± 1.00	5.38 ± 1.02	5.38 ± 1.53	χ^2(2, N=63)=7.84, p<.05*	Jibo vs Alexa: Z=2.23, p<.05*<br>Jibo vs Google: Z=1.59, p>.05
Anxiously wanting to engage with me	5.29 ± 2.19	3.76 ± 1.92	3.62 ± 1.86	χ^2(2, N=63)=20.11, p<1e-04****	Jibo vs Alexa: Z=3.22, p<.01**<br>Jibo vs Google: Z=2.99, p<.01**
Consistent & predictable	5.05 ± 1.88	4.95 ± 2.11	4.33 ± 1.96	χ^2(2, N=63)=2.25, p>.05	N/A
Confused at times and may mess up	3.95 ± 2.27	3.81 ± 2.02	4.67 ± 1.91	χ^2(2, N=63)=1.49, p>.05	N/A
Sympathetic & warm	5.86 ± 1.80	4.19 ± 2.06	3.67 ± 1.85	χ^2(2, N=63)=20.10, p<1e-04****	Jibo vs Alexa: Z=3.13, p<.01**<br>Jibo vs Google: Z=3.54, p<.001***
Opinionated and shares its thoughts	5.71 ± 1.71	4.19 ± 1.66	4.05 ± 1.50	χ^2(2, N=63)=18.70, p<1e-04****	Jibo vs Alexa: Z=3.30, p<.001***<br>Jibo vs Google: Z=3.26, p<.01**
Always learning about me	4.81 ± 2.69	3.43 ± 2.40	3.62 ± 2.33	χ^2(2, N=63)=9.91, p<.01**	Jibo vs Alexa: Z=2.68, p<.01**<br>Jibo vs Google: Z=2.73, p<.01**

Table S2. Comparing perceived personality traits across the three VUI agents in Study 1. There was significant difference between the agents for the “outgoing and engages me lots”, “simple in personality”, “always learning about me”, “opinionated and shares its thoughts”, “sympathetic and warm” , “anxiously wanting to engage with me”, and “dependable and tries to help me” traits.

**User Experience Factor**	**Jibo** mean±std	**Alexa** mean±std	**Google** mean±std	**Friedman test**	**Post-hoc Wilcoxon with Holm correction**
Perceived trust	2.29 ± 1.08	1.80 ± 1.11	1.71 ± 1.09	χ^2(2, N=492)= 40.07, p<1e-04****	Jibo vs Alexa: Z=4.68, p<1e-04****<br>Jibo vs Google: Z=4.97, p<1e-04****
Perceived companionship	2.60 ± 0.99	1.07 ± 0.88	0.97 ± 0.80	χ^2(2, N=180)= 81.45, p<1e-04****	Jibo vs Alexa: Z=6.05, p<1e-04****<br>Jibo vs Google: Z=6.30, p<1e-04****
Perceived competence	2.31 ± 0.93	1.93 ± 1.03	1.87 ± 0.97	χ^2(2, N=507)=17.13, p<.001***	Jibo vs Alexa: Z=3.60, p<.001***<br>Jibo vs Google: Z=4.10, p<1e-04****
Perceived emotional engagement	2.62 ± 0.87	1.37 ± 0.88	1.32 ± 0.82	χ^2(2, N=891)= 320.32, p<1e-04****	Jibo vs Alexa: Z=12.61, p<1e-04****<br>Jibo vs Google: Z=12.97, p<1e-04****

Table S3. Comparing perception of trust, companionship, competence, and emotional engagement around the three VUI agents in Study 1. The perceived trust, companionship, competence, and emotional engagement showed statistically significant difference between the agents are highlighted.

2 STUDY 2 DETAILED RESULTS

Interaction Type	Jibo mean±std	Amazon mean±std	Google mean±std	Friedman test	Post-hoc Wilcoxon with Holm correction
Total Interaction	15.09 ± 12.02	4.91 ± 3.64	4.19 ± 3.38	χ^2(2, N=96)=21.39, p<1e-04****	Jibo vs Amazon: Z=4.20, p<1e-04****<br>Jibo vs Google: Z=4.47, p<1e-04****
Interpersonal	9.25 ± 8.87	2.41 ± 2.09	2.50 ± 2.03	χ^2(2, N=96)=23.03, p<1e-04****	Jibo vs Amazon: Z=4.04, p<1e-04****<br>Jibo vs Google: Z=4.28, p<1e-04****
Entertainment	3.25 ± 2.57	1.84 ± 2.32	1.03 ± 0.97	χ^2(2, N=96)=14.40, p<.001***	Jibo vs Amazon: Z=2.31, p<.05*<br>Jibo vs Google: Z=4.00, p<1e-04****
Information	2.59 ± 2.70	0.66 ± 0.87	0.66 ± 0.97	χ^2(2, N=96)=16.90, p<0.001***	Jibo vs Amazon: Z=3.43, p<.001***<br>Jibo vs Google: Z=3.53 , p<.001***

Table S4. Comparing interaction types around the three VUI agents in Study 2. The overall, interpersonal, entertainment, and information interactions showed statistically significant difference between the agents are highlighted.

Personality Trait	Jibo mean±std	Amazon mean±std	Google mean±std	Friedman test	Post-hoc Wilcoxon with Holm correction
Simple in personality	1.52 ± 1.31	4.74 ± 2.05	4.67 ± 2.04	χ^2(2, N=81)=31.09, p<1e-04****	Jibo vs Amazon: Z=3.77, p<.001***<br>Jibo vs Google: Z=4.31, p<1e-04****
Quiet & keeps to itself in personality	2.96 ± 2.14	5.07 ± 1.69	5.11 ± 2.03	χ^2(2, N=81)=18.09, p<.001***	Jibo vs Amazon: Z=3.58, p<.001***<br>Jibo vs Google: Z=2.95, p<.01**
Outgoing & engages me lots	6.63 ± 0.69	3.96 ± 1.83	3.96 ± 1.56	χ^2(2, N=81)=39.03, p<1e-04****	Jibo vs Amazon: Z=4.36, p<1e-04****<br>Jibo vs Google: Z=4.56, p<1e-04****
Dependable & tries to help me	5.96 ± 1.89	5.04 ± 2.14	5.44 ± 1.95	χ^2(2, N=81)=6.57, p<.05*	Jibo vs Amazon: Z=2.04, p<.05*<br>Jibo vs Google: Z=1.58, p>.05
Anxiously wanting to engage with me	4.70 ± 2.28	3.00 ± 2.13	3.19 ± 2.22	χ^2(2, N=81)=22.04, p<1e-04****	Jibo vs Amazon: Z=3.19, p<.01**<br>Jibo vs Google: Z=3.19, p<.01**
Consistent & predictable	3.81 ± 2.39	3.04 ± 1.97	3.30 ± 2.09	χ^2(2, N=81)=3.48, p>.05	N/A
Confused at times and may mess up	4.48 ± 2.34	4.37 ± 1.71	3.96 ± 2.01	χ^2(2, N=81)=7.85, p<.05*	Jibo vs Amazon: Z=0.59, p>.05<br>Jibo vs Google: Z=1.70, p>.05
Sympathetic & warm	6.56 ± 0.93	4.11 ± 1.95	4.19 ± 2.06	χ^2(2, N=81)=34.60, p<1e-04****	Jibo vs Amazon: Z=4.03, p<1e-04****<br>Jibo vs Google: Z=4.03, p<1e-04****
Opinionated and shares its thoughts	5.63 ± 1.84	4.11 ± 2.14	4.33 ± 2.29	χ^2(2, N=81)=16.66, p<.001***	Jibo vs Amazon: Z=2.88, p<.01**<br>Jibo vs Google: Z=2.51, p<.05*
Always learning about me	4.67 ± 2.48	3.22 ± 2.31	3.07 ± 2.20	χ^2(2, N=81)=23.67, p<1e-04****	Jibo vs Amazon: Z=3.31, p<.001***<br>Jibo vs Google: Z=3.44, p<.001***

Table S5. Comparing perceived personality traits across the three VUI agents in Study 2. There was significant difference between the agents for the “outgoing and engages me lots”, “simple in personality”, “always learning about me”, “opinionated and shares its thoughts”, “sympathetic and warm” , “anxiously wanting to engage with me”, “confused and may mess up”, “quiet and keeps to itself”, and “dependable and tries to help me” traits.

**User Experience Factor**	**Jibo** mean±std	**Amazon** mean±std	**Google** mean±std	**Friedman test**	**Post-hoc Wilcoxon with Holm correction**
Perceived trust	2.28 ± 1.09	1.44 ± 1.02	1.60 ± 1.10	χ^2(2, N=600)=85.39, p<1e-04****	Jibo vs Amazon: Z=7.43, p<1e-04****<br>Jibo vs Google: Z=6.08, p<1e-04****<br>Amazon vs Google: Z=3.27, p<.01**
Perceived companionship	2.47 ± 1.14	0.88 ± 0.66	1.08 ± 0.80	χ^2(2, N=225)=107.37, p<1e-04****	Jibo vs Amazon: Z=7.15, p<1e-04****<br>Jibo vs Google: Z=7.00, p<1e-04****<br>Amazon vs Google: Z=2.61, p<.01**
Perceived competence	2.43 ± 0.97	1.47 ± 0.98	1.74 ± 1.05	χ^2(2, N=597)=92.46, p<1e-04****	Jibo vs Amazon: Z=7.70, p<1e-04****<br>Jibo vs Google: Z=5.65, p<1e-04****<br>Amazon vs Google: Z=3.40, p<.001***
Perceived emotional engagement	2.76 ± 0.76	1.05 ± 0.77	1.30 ± 0.91	χ^2(2, N=1059)=473.20, p<1e-04****	Jibo vs Amazon: Z=15.19, p<1e-04****<br>Jibo vs Google: Z=14.14, p<1e-04****<br>Amazon vs Google: Z=5.55, p<1e-04****

Table S6. Comparing perception of trust, companionship, competence, and emotional engagement around the three VUI agents in Study 2. The perceived trust, companionship, competence, and emotional engagement showed statistically significant difference between the agents are highlighted.

3 STUDY 3 DETAILED RESULTS

Interaction Type	Jibo mean±std	Alexa mean±std	Computer mean±std	Friedman test	Post-hoc Wilcoxon with Holm correction
Total Interaction	7.36 ± 5.47	4.64 ± 3.47	4.0 ± 3.09	χ^2(2, N=99)=14.19, $p<.001^{***}$	Jibo vs Alexa: Z=3.66, $p<.001^{***}$<br>Jibo vs Comp.: Z=3.58, $p<.001^{***}$
Interpersonal	4.06 ± 3.45	2.45 ± 2.11	2.0 ± 1.67	χ^2(2, N=93)=7.10, $p<.05^{*}$	Jibo vs Alexa: Z=2.93, $p<.01^{**}$<br>Jibo vs Comp.: Z=2.88, $p<.01^{**}$
Entertainment	1.87 ± 1.33	1.13 ± 1.23	1.0 ± 1.15	χ^2(2, N=93)=13.83, $p<.001^{***}$	Jibo vs Alexa: Z=3.07, $p<.01^{**}$<br>Jibo vs Comp.: Z=2.78, $p<.01^{**}$
Information	1.71 ± 1.42	1.29 ± 1.27	1.23 ± 1.02	χ^2(2, N=93)=5.04, $p>.05$	N/A

Table S7. Comparing interaction types around the three VUI agents in Study 3. The overall, interpersonal, and entertainment interactions showed statistically significant difference between the agents are highlighted.

Personality Trait	Jibo mean±std	Alexa mean±std	Computer mean±std	Friedman test	Post-hoc Wilcoxon with Holm correction
Simple in personality	3.81 ± 2.30	5.0 ± 1.51	5.30 ± 1.45	χ^2(2, N=111)=9.09, $p<.05^{*}$	Jibo vs Alexa: Z=2.58, $p<.001^{***}$<br>Jibo vs Comp.: Z=2.56, $p<.05^{*}$<br>Alexa vs Comp.: Z=0.75, $p>.05$
Quiet & keeps to itself in personality	2.78 ± 2.02	4.14 ± 1.80	5.65 ± 1.74	χ^2(2, N=111)=25.33, $p<.001^{***}$	Jibo vs Alexa: Z=3.45, $p<.001^{***}$<br>Jibo vs Comp.: Z=4.26, $p<$1e-04****<br>Alexa vs Comp.: Z=3.33, $p<.001^{***}$
Outgoing & engages me lots	6.32 ± 1.25	4.35 ± 1.30	3.14 ± 1.70	χ^2(2, N=111)=55.20, $p<$1e-04****	Jibo vs Alexa: Z=4.61, $p<$1e-04****<br>Jibo vs Comp.: Z=4.75, $p<$1e-04****<br>Alexa vs Comp.: Z=3.26, $p<.01^{**}$
Dependable & tries to help me	5.65 ± 1.46	5.16 ± 1.24	4.41 ± 1.64	χ^2(2, N=111)=20.33, $p<$1e-04****	Jibo vs Alexa: Z=2.07, $p<.05^{*}$<br>Jibo vs Comp.: Z=3.22, $p<.01^{**}$<br>Alexa vs Comp.: Z=3.37, $p<.001^{***}$
Anxiously wanting to engage with me	5.65 ± 1.51	3.81 ± 1.68	3.0 ± 1.56	χ^2(2, N=111)=44.99, $p<$1e-04****	Jibo vs Alexa: Z=4.28, $p<$1e-04****<br>Jibo vs Comp.: Z=4.47, $p<$1e-04****<br>Alexa vs Comp.: Z=2.86, $p<.01^{**}$
Consistent & predictable	4.70 ± 1.56	5.19 ± 1.35	5.14 ± 1.49	χ^2(2, N=111)=4.67, $p>.05$	N/A
Confused at times and may mess up	4.16 ± 1.86	3.84 ± 1.82	4.41 ± 1.64	χ^2(2, N=111)=0.93, $p>.05$	N/A
Sympathetic & warm	6.14 ± 1.34	3.78 ± 1.78	2.84 ± 1.82	χ^2(2, N=111)=42.48, $p<$1e-04****	Jibo vs Alexa: Z=4.44, $p<$1e-04****<br>Jibo vs Comp.: Z=4.75, $p<$1e-04****<br>Alexa vs Comp.: Z=2.65, $p<.01^{**}$
Opinionated and shares its thoughts	5.76 ± 1.50	4.0 ± 1.60	3.03 ± 1.44	χ^2(2, N=111)=44.66, $p<$1e-04****	Jibo vs Alexa: Z=4.33, $p<$1e-04****<br>Jibo vs Comp.: Z=4.95, $p<$1e-04****<br>Alexa vs Comp.: Z=2.72, $p<.01^{**}$
Always learning about me	5.22 ± 1.55	4.32 ± 1.36	3.24 ± 1.26	χ^2(2, N=111)=29.81, $p<$1e-04****	Jibo vs Alexa: Z=2.52, $p<.05^{*}$<br>Jibo vs Comp.: Z=4.11, $p<$1e-04****<br>Alexa vs Comp.: Z=3.74, $p<.001^{***}$

Table S8. Comparing perceived personality traits across the three VUI agents in Study 3. There was significant difference between the agents for the “outgoing and engages me lots”, “simple in personality”, “always learning about me”, “opinionated and shares its thoughts”, “sympathetic and warm” , “anxiously wanting to engage with me”, “quiet and keeps to itself”, and “dependable and tries to help me” traits.

**User Experience Factor**	**Jibo** mean±std	**Alexa** mean±std	**Computer** mean±std	**Friedman test**	**Post-hoc Wilcoxon with Holm correction**
Perceived trust	2.05 ± 1.23	1.80 ± 1.20	1.48 ± 1.15	χ^2(2, N=888)=95.45, $p<$1e-04****	Jibo vs Alexa: Z=4.01, $p<$1e-04****<br>Jibo vs Comp.: Z=7.87, $p<$1e-04****<br>Alexa vs Comp.: Z=6.04, $p<$.01**
Perceived companionship	2.0 ± 1.46	1.10 ± 1.0	0.83 ± 0.85	χ^2(2, N=333)=119.42, $p<$1e-04****	Jibo vs Alexa: Z=7.15, $p<$1e-04****<br>Jibo vs Comp.: Z=7.43, $p<$1e-04****<br>Alexa vs Comp.: Z=5.00, $p<$.01**
Perceived competence	2.19 ± 1.12	1.87 ± 1.06	1.58 ± 1.07	χ^2(2, N=888)=79.77, $p<$1e-04****	Jibo vs Alexa: Z=4.34, $p<$1e-04****<br>Jibo vs Comp.: Z=7.23, $p<$1e-04****<br>Alexa vs Comp.: Z=4.40, $p<$1e-04****
Perceived emotional engagement	2.35 ± 1.15	1.47 ± 0.96	1.11 ± 0.87	χ^2(2, N=1554)=521.51, $p<$1e-04****	Jibo vs Alexa: Z=15.49, $p<$1e-04****<br>Jibo vs Comp.: Z=16.66, $p<$1e-04****<br>Alexa vs Comp.: Z=9.17, $p<$1e-04****

Table S9. Comparing perception of trust, companionship, competence, and emotional engagement around the three VUI agents in Study 3. The perceived trust, companionship, competence, and emotional engagement showed statistically significant difference between the agents are highlighted.
